# Web-Based Video-Coaching to Assist an Automated Computer-Tailored Physical Activity Intervention for Inactive Adults: A Randomized Controlled Trial

**DOI:** 10.2196/jmir.5664

**Published:** 2016-08-12

**Authors:** Stephanie Alley, Cally Jennings, Ronald C Plotnikoff, Corneel Vandelanotte

**Affiliations:** ^1^ Physical Activity Research Group School of Human, Health and Social Sciences Central Queensland University Rockhampton Australia; ^2^ Alberta Center for Active Living Faculty of Physical Education and Recreation University of Alberta Edmonton, AB Canada; ^3^ Priority Research Centre for Physical Activity and Nutrition Faculty of Health and Medicine University of Newcastle Callaghan Australia

**Keywords:** motor activity, health promotion, chronic disease, e-counseling, Internet

## Abstract

**Background:**

Web-based physical activity interventions that apply computer tailoring have shown to improve engagement and behavioral outcomes but provide limited accountability and social support for participants. It is unknown how video calls with a behavioral expert in a Web-based intervention will be received and whether they improve the effectiveness of computer-tailored advice.

**Objective:**

The purpose of this study was to determine the feasibility and effectiveness of brief video-based coaching in addition to fully automated computer-tailored advice in a Web-based physical activity intervention for inactive adults.

**Methods:**

Participants were assigned to one of the three groups: (1) tailoring + video-coaching where participants received an 8-week computer-tailored Web-based physical activity intervention (“My Activity Coach”) including 4 10-minute coaching sessions with a behavioral expert using a Web-based video-calling program (eg, Skype; n=52); (2) tailoring-only where participants received the same intervention without the coaching sessions (n=54); and (3) a waitlist control group (n=45). Demographics were measured at baseline, intervention satisfaction at week 9, and physical activity at baseline, week 9, and 6 months by Web-based self-report surveys. Feasibility was analyzed by comparing intervention groups on retention, adherence, engagement, and satisfaction using *t* tests and chi-square tests. Effectiveness was assessed using linear mixed models to compare physical activity changes between groups.

**Results:**

A total of 23 tailoring + video-coaching participants, 30 tailoring-only participants, and 30 control participants completed the postintervention survey (83/151, 55.0% retention). A low percentage of tailoring + video-coaching completers participated in the coaching calls (11/23, 48%). However, the majority of those who participated in the video calls were satisfied with them (5/8, 71%) and had improved intervention adherence (9/11, 82% completed 3 or 4 modules vs 18/42, 43%, *P*=.01) and engagement (110 minutes spent on the website vs 78 minutes, *P*=.02) compared with other participants. There were no overall retention, adherence, engagement, and satisfaction differences between tailoring + video-coaching and tailoring-only participants. At 9 weeks, physical activity increased from baseline to postintervention in all groups (tailoring + video-coaching: +150 minutes/week; tailoring only: +123 minutes/week; waitlist control: +34 minutes/week). The increase was significantly higher in the tailoring + video-coaching group compared with the control group (*P*=.01). No significant difference was found between intervention groups and no significant between-group differences were found for physical activity change at 6 months.

**Conclusions:**

Only small improvements were observed when video-coaching was added to computer-tailored advice in a Web-based physical activity intervention. However, combined Web-based video-coaching and computer-tailored advice was effective in comparison with a control group. More research is needed to determine whether Web-based coaching is more effective than stand-alone computer-tailored advice.

**Trial Registration:**

Australian New Zealand Clinical Trials Registry (ACTRN): 12614000339651; http://www.anzctr.org.au/TrialSearch.aspx?searchTxt=ACTRN12614000339651+&isBasic=True (Archived by WebCite at http://www.webcitation.org/6jTnOv0Ld)

## Introduction

Physical activity improves physical and mental health and significantly lowers the risk of noncommunicable disease, including cardiovascular disease, diabetes, and cancer [1]. Australian guidelines recommend 150-300 minutes of moderate-intensity activity each week, over 5 days, to receive health benefits and reduce the risk of noncommunicable disease [2]. Despite this, less than 50% of Australians meet these recommendations [3]. As such, there is a need for effective and affordable physical activity interventions with a broad reach.

Health behavior change interventions delivered via the Web have the potential to reach a large audience at low cost, due to the majority of Australians (92%) having access to the Web [4]. Furthermore, they are convenient for participants and enable the content to be delivered in a nonconfrontational manner [5-7]. Many studies have found Web-based physical activity interventions to be effective in the short term. For example, reviews of Web-based physical activity interventions have found more than half of controlled trials to report positive physical activity outcomes [8,9]. However, problems with low engagement and high dropout rates lead to small and short-term behavior changes [8,10]. Furthermore, many interventions struggle to reach those most in need of increasing their physical activity [8,10-12]. Personalized health advice through coaching sessions or computer-tailored feedback engages participants and improves the effectiveness of Web-based health behavior interventions [13-15]. Both Web-based coaching sessions and computer-tailored advice provide support similar to traditional face-to-face coaching sessions, at a lower cost with fewer geographical restrictions [13,16].

Coaching is defined as facilitating health behavior change through interactions between a health professional (coach) and a client [17]. Web-based coaching sessions are most similar to traditional face-to-face coaching as they provide personal interaction. Coaching in Web-based physical activity interventions improves participants’ perceptions of their social support, which is associated with greater levels of behavior change [18,19]. The Social Cognitive Theory stipulates that the acquisition of a new behavior is influenced by the individuals’ cognitive factors (eg, attitude), behavioral factors (eg, skills), and contextual factors including reinforcement, instructions, and social norms [20]. Including coaching in a Web-based intervention addresses the otherwise overlooked social contextual factors that play an important role in behavior change [20].

Advances in Internet technology and broadband capacity now allow the option of delivering coaching sessions via free Web-based video-calling programs, which allow participants to view the coach and engage in a verbal discussion. The popular video-calling programs Skype, Google Hangouts, and FaceTime are encrypted, which ensures privacy of participant information [21]. Video-coaching facilitates higher engagement, feelings of accountability, and social support, and reduces the risk of misunderstandings compared with emails and instant messaging [22,23]. Video-coaching has been found to be effective in producing changes in parenting behavior [24], to have a high feasibility for smoking cessation [25], and a high feasibility for supporting in-home rehabilitation in the elderly [26]. However, no studies have used video-coaching as part of a Web-based physical activity program. Despite the potential of Web-based video-coaching, its reliance on the time of a behavior change expert leads to higher implementation costs compared with fully automated computer-tailored advice.

Computer tailoring can deliver personalized advice at a low cost by using a computer-based expert system to automatically deliver feedback to participants’ responses to a Web-based questionnaire [13]. Computer-tailored physical activity advice is preferred by participants, leads to greater attention [27], and improved health behavior outcomes compared with generic health advice [14]. Although the effectiveness of computer tailoring is well established and it has the benefit of providing personalized advice to large numbers at low cost, it is unknown whether it could be more effective with an element of human support.

To our knowledge, no health behavior interventions have combined computer-tailored advice with Web-based video-coaching. This approach may improve intervention outcomes by utilizing the benefits of both methods. Providing computer-tailored advice can limit the time required from a video-coach, therefore limiting costs, as well as reducing reliance on the knowledge and expertise of the coach. A brief coaching session can add an element of social support, as well as further explanation, personalization, and interpretation of theory-based computer-tailored advice received by participants at an earlier time. It is unknown whether brief video-coaching sessions to reiterate computer-tailored physical activity advice are feasible in terms of retention, adherence, engagement, and satisfaction. It is also unknown whether they lead to improved physical activity and quality of life compared with stand-alone computer-tailored advice. Therefore, this study explores the feasibility and effectiveness of a brief Web-based coaching session in addition to computer-tailored advice for inactive adults.

The first aim of the study was to determine the feasibility of brief video-coaching, when used to discuss previously received computer-tailored physical activity advice, in a stand-alone Web-based intervention for inactive adults. Feasibility was determined by adherence and satisfaction of the coaching sessions and comparing intervention retention, adherence, website engagement, and satisfaction of the tailoring + video-coaching and tailoring-only groups. The second aim was to test the effectiveness of the video-coaching sessions in terms of physical activity and quality of life outcomes. It was hypothesized that computer tailoring in combination with video-coaching would result in greater retention, adherence, engagement, and satisfaction with the intervention, compared with a computer-tailored–only group and a waitlist control group. It was also hypothesized that computer tailoring and video-coaching would result in greater improvements in quality of life and physical activity compared with a computer-tailored–only group and a waitlist control group.

## Methods

### Research Procedure

A detailed account of the methods can be found in the protocol paper [28] and a consort eHealth checklist for the paper can be found here (Multimedia Appendix 1) [29]. The recruitment methods, participant eligibility, protocol, intervention description, measures, and data analysis are summarized below.

### Recruitment

Print advertising and Web advertising were used to recruit participants from a number of Australian metropolitan and regional cities (Sydney, Melbourne, Perth, Brisbane, Rockhampton, Bundaberg, Mackay, and Townsville). Print advertising included newspaper advertisements and articles, posters and leaflets displayed in health clinics, and leaflets distributed to peoples’ homes. The Web advertising included links displayed on community websites and paid advertisements on Google and Facebook. Ethics approval was received from the Central Queensland University Human Research Ethics Committee (H13/04-044), before recruitment took place from March 2014 to January 2015. This study is registered with the Australian New Zealand Clinical Trials Registry (ACTRN12614000339651).

### Participants

People were eligible to participate if they were English-speaking Australian adults (older than 18 years). Participants were excluded if they were pregnant, at risk of injury or ill health from their increasing physical activity (as assessed by the Physical Activity Readiness Questionnaire), or if they were already meeting the physical activity recommendations (as assessed via a single item asking if participants participated in 30 minutes of physical activity on most days). It is likely that the intervention attracted participants with a high Internet literacy.

### Protocol

Information about the study, including the affiliation with Central Queensland University, was available on the landing page of the intervention website (Multimedia Appendices 2 and 3). To assess individuals’ eligibility, how they heard about the program, and collect contact details, a screening questionnaire was delivered through the intervention website. Eligible participants were randomly assigned based on a sequence (not concealed) of random numbers between 1 and 3 to one of three study arms: tailoring + video-coaching, tailoring-only, or waitlist control. This was done in blocks of 15 participants. SA generated the random allocation sequence and assigned participants to groups. Participants remained blinded to their condition until after completing all baseline measures. Participants began the intervention on the Monday following their recruitment. The consent form and then baseline questionnaire were administered through the intervention website for all groups. Upon completing the baseline questionnaire, the intervention groups received module 1 of their personalized advice, whereas the control group received nothing. The intervention “My Activity Coach” delivered 1 module of computer-tailored advice every 2 weeks over 8 weeks (4 modules in total). During the weeks where no new modules were received, participants in the tailoring + video-coaching group received a brief coaching session through a Web-based video-calling program (eg, Skype) to reiterate the advice received in their previous module. Participants in the tailoring-only group received an email reminding them of the tailored advice they received in the previous module to ensure both intervention groups received the same number of contacts. Participants in the waitlist control group were given the opportunity to participate in the intervention without coaching after they completed the final questionnaire. Questionnaires were administered through the intervention website immediately after the end of the intervention (week 9) and 6 months after the end of the intervention. Participants had to log in to complete each survey, which ensured they were only completed once. The week 9 questionnaires were collected from June 2014 to March 2015. It was not possible to blind researchers to participants’ group assignment after they had completed the baseline questionnaire. Participants were blinded to their group assignment only when completing their baseline questionnaire. The consent sheet explained the 2 interventions and therefore it is possible that participants worked out whether they were in the intervention of interest or comparator. Participants who completed all surveys went in the draw to win 1 of 30 pedometers, 6 Fitbits, and 3 heart rate monitors. Because many individuals began but failed to complete the screening questionnaire, we tested conducting the screening questionnaire by phone (after receiving an ethics amendment from the Central Queensland University’s Human Research Ethics Committee). This was done for 15 prospective participants and discontinued because of failure to increase screening completions. The Web-based booking system for coaching participants was changed halfway through the trial as the first booking system was discontinued. No other changes to the protocol were carried out during the trial.

### Intervention

The 8-week “My Activity Coach” Web-based intervention delivered a new module of tailored advice to participants every 2 weeks [30]. Each module required participants to complete a brief Web-based questionnaire about their physical activity and psychosocial correlates of physical activity. Feedback was then provided based on their responses to the questionnaire (Multimedia Appendices 4 and 5). Participants received up to 4 reminder emails and a reminder phone call when they did not complete the survey required for each module. The tailored advice was based on behavior change theory (Theory of Planned Behavior [31]) and communication theory (Elaboration Likelihood Model [32]). Each module began with a graph including bars to represent participants’ current physical activity, their physical activity during the previous modules, as well as the minimum and optimal physical activity recommendations. Module 1, titled “Are you active enough,” explained the physical activity recommendations and health benefits of physical activity tailored to their body mass index (BMI), age, and level of physical activity. The module ended with a suggested goal (based on their current activity level) to work toward until the next module. Module 2, titled “Let’s set some goals,” provided participants with information on goal setting and action planning. Module 3, titled “Physical activity and your environment,” delivered tailored information on using participants’ social and physical environments to increase their physical activity. Module 4, titled “Staying active,” addressed relapse prevention. Participants also received tailored advice on their perceived benefits and barriers to being active and self-efficacy to become more active throughout the modules. The modules and intervention website were adapted from an earlier 2-module Web-based intervention with computer-tailored advice for inactive adults [33]. Focus groups were conducted to inform development of this prior intervention [34]. Updates were conducted by a website developer who also created the original intervention website.

An action-planning tool became available to participants after module 2. The tool allowed participants to create an action plan for up to 4 activities (specifying where, when, for how long, and with whom they will be active over the following 2 weeks). Participants could print a calendar-based overview of their action plan (Multimedia Appendix 6). The coaching group’s 10- to 15-minute biweekly video-coaching sessions were conducted through a Web-based video-calling program of participants’ choice (eg, Skype, Google Hangouts, Yahoo Messenger, and FaceTime). During the session the activity coach commented on the tailored advice participants received in the module from the previous week, answered any questions participants had, and provided encouragement, support, and accountability.

### Measures

Participants’ demographics including sex, age, BMI, household income (less than AUD $31,200, AUD $31,200-$77,999, more than AUD $78,000), education (less than secondary, secondary, further education), and employment (full time, part time or casual, and not in paid employment) were assessed in the baseline survey. Participants were also asked if they used a video-calling program (Skype, Google Hangouts, FaceTime, other, none). Completion of the coaching sessions, the length of the coaching sessions, and reasons for missed coaching sessions were recorded by the coach. Intervention retention and adherence were assessed by recording participants’ completion of the research surveys and intervention modules, respectively, and website engagement was measured through Google Analytics. Google Analytics recorded the number of website visits and time spent on the website for each participant. Intervention participants’ satisfaction with the intervention was assessed at the end of the intervention (week 9). Satisfaction with module questions (4 items), computer-tailored advice (14 items), website usability (13 items), overall program (5 items), and coaching (14 items) were all assessed. The items were specifically developed for this study, although based on previous research [35]. The items were on a 5-point Likert scale where participants were asked to rate their agreement (1=strongly agree to 5= strongly disagree) to statements about the intervention. All positively framed questions were reverse scored. For each category participants with a mean rating of 3.6 or higher (maximum = 5) were categorized as “satisfied.” Coaching participants were also asked if they completed a coaching session and if not, why not. All intervention participants were asked 4 open-ended questions about 3 topics: the advice, website, and overall program. Coaching participants who completed a coaching session were asked an additional 4 open-ended questions specifically relating to the coaching sessions. The 4 questions for each category (advice, website, program, and coaching) were (1) what did you like about the advice, website, program, or coaching; (2) what did you not like; (3) any recommendations for improvement; and (4) any other thoughts (Multimedia Appendix 7). Responses for all questions were thematically analyzed. The main outcome, weekly physical activity, was assessed at all time points via the Active Australia Survey, (AAS), which has a high percentage agreement with other physical activity measures (67%-75%) [36] and has a good test-retest reliability (kappa = .52) [37] including when self-administered [38]. Quality of life was measured at all 3 time points by the SF-12 version 2, which is valid [39] and reliable [40] including when self-administered on the Web [41]. Physical health and mental health component scores were calculated from the SF-12 version 2 following manual instructions [42].

### Data Analysis

Baseline demographics for participants in each trial arm (tailoring + video-coaching, tailoring-only, and control) are presented (Table 1). The demographics of completers versus dropouts, as well as coaching participants who did versus did not complete a coaching session, were compared using chi-square and *t* tests. To test feasibility of the coaching sessions, completion of the coaching sessions, the length of the coaching sessions, and reasons for missed coaching sessions were presented. Next, the 2 intervention groups, as well as coaching participants who did versus did not complete a coaching session, were compared on retention (dropouts vs completers) and adherence (completed 1-2 modules vs 3-4 modules) using a chi-square test, number of website visits and time spent on the website using *t* tests, and satisfaction scores (satisfied vs neutral or not satisfied) using a chi-square test. To test effectiveness, longitudinal data were analyzed using intention-to-treat principles. Physical activity, mental health score, and physical health score were each modeled using linear mixed models with time (baseline, week 9, and 6 months) as a repeated factor, fixed effects of time and group (control, tailoring-only, tailoring + video-coaching), and a time by group interaction (Table 2). Significance level was set to *P*<.05.

### Sample Size

Sample size calculations demonstrated that a sample size of 300, or 100 in each study arm, was required to detect between-group differences in physical activity from baseline to postintervention using linear mixed models [43]. This calculation was based on the alpha level of ≤.05 (80% power) and a small effect size (0.43) and 25% attrition, which are common in similar interventions [10].

## Results

### Flow of Participants

**Figure 1:**
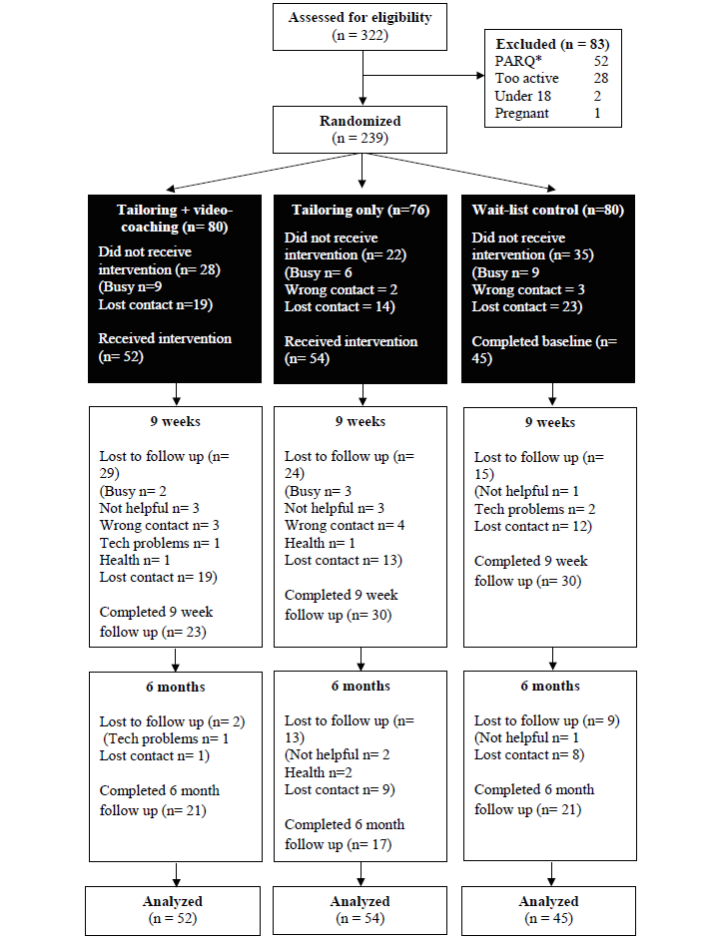
Participant flow through the intervention trial. *PARQ: Physical Activity Readiness Questionnaire.

Of the 239 randomly assigned participants, 154 completed the baseline questionnaire and at least one of the intervention modules. Of these, 84 participants completed the postintervention survey at week 9 (55% retention). A total of 59 participants completed the 6-month follow-up questionnaire (38% retention). There were no demographic differences between those who completed the week 9 survey and those who did not. The majority of participants were recruited through Facebook (63%), and small percentages were recruited through Google (8%), a newspaper article (6%), letterbox drops (5%), family or friend (5%), leaflets (5%), posters (4%), community websites (3%), and newspaper advertisements (1%). Figure 1 presents the flow of participants through the trial.

**Table 1:** Baseline characteristics, physical activity, and quality of life by group assignment

Variables		Tailoring + video coaching	Tailoring only	Control
**Sex n=154, n (%)**				
	Male	16 (30)	14 (25)	7 (16)
	Female	37 (67)	42 (75)	38 (84)
**Employment n=151^a^, n (%)**				
	Full time	19 (37)	21 (38)	18 (40)
	Part time or casual	10 (19)	10 (19)	9 (20)
	Not in paid employment	23 (44)	23 (43)	18 (40)
**Education n=151^a^, n (%)**				
	Less than secondary	2 (4)	0 (0)	0 (0)
	Secondary	8 (15)	10 (19)	6 (13)
	Further education	42 (81)	44 (81)	39 (87)
**Income n=112^b^, n (%)**				
	More than AUD $78,000	21 (55)	14 (36)	21 (60)
	AUD $31,200-$77,999	11 (29)	17 (44)	6 (17)
	Less than AUD $31,199	6 (16)	8 (20)	8 (23)
**Uses Web-based video-calling n=151^a^, n (%)**				
	Yes	51 (63)	53 (68)	63 (79)
	No	30 (37)	25 (32)	17 (21)
Age (years), n=154		55.26 (10.93)	52.18 (11.53)	55.18 (13.45)
BMI^c^, n=150^d^, M(SD)		32.08 (7.43)	31.58 (7.43)	29.97 (6.75)
Baseline total physical activity (minutes/week), n=151, M(SD)		189.52 (214.30)	152.87 (174.33)	160.44 (191.23)
Week 9 total physical activity (minutes/week), n=83, M(SD)		387.83 (264.89)	315.17 (264.39)	211.00 (164.16)
6-month total physical activity (minutes/week), n=59, M(SD)		419.52 (77.109)	319.71 (164.77)	305.00 (315.86)
Baseline mental health score, n=148, M(SD)		46.10 (1.30)	41.36 (12.27)	42.74 (1.78)
Week 9 mental health score, n=82, M(SD)		48.97 (10.27)	30.03 (6.12)	45.19 (10.50)
6-month mental health score, n=59, M(SD)		48.16 (12.11)	43.90 (10.34)	44.94 (11.84)
Baseline physical health score, n=146, M(SD)		46.90 (10.17)	51.92 (8.28)	48.62 (8.98)
Week 9 physical health score, n=78, M(SD)		46.88 (12.05)	51.48 (6.95)	47.32 (9.37)
6-month physical health score, n=59, M(SD)		46.60 (10.22)	52.38 (5.47)	48.18 (10.46)

^a^Baseline data (employment, education, video calling use and BMI) were lost for 3 participants.

^b^A total of 39 respondents chose not to disclose their income.

^c^BMI: body mass index.

^d^ BMI was lost for four participants.

### Sample Characteristics

The majority of participants were female (117/154, 76.0%) and were on average 54 years of age. Just under half (64/151, 43%) were not in paid employment and the majority (125/151, 82.8%) had completed a higher education course. Less than half (n=60/151, 39.7%) of participants were physically active and on average participated in 168 minutes of physical activity. The average BMI was 31, which is in the obese range. The majority (n=106/151, 70.2%) used a video-calling program.

### Coaching Adherence and Satisfaction

Coaching participants had low adherence to the coaching sessions. Just under half of coaching group completers (11/23, 48%) and less than a quarter of all coaching participants (11/52, 21%) completed 1 coaching session. There were no demographic differences between those who participated in a coaching session and those who did not. Of the coaching participants who did not do a coaching session, 8 wanted a second chance to do a session but did not book or show up the second time, 7 refused to do one, 4 were too busy, 7 had technical difficulties, 2 had injuries, and contact was lost with 13. Of those who participated in at least 1 coaching session, an average of 2.4 sessions were completed, the average coaching session length was 10.4 minutes, and 15% of the coaching calls were interrupted with technical difficulties. A total of 8 coaching participants (8/52, 15%) completed the coaching satisfaction questions. The majority were satisfied with the coaching sessions (n=5/8, 71.4%). In response to open-ended coaching questions, participants said the sessions held them accountable (n=3), appreciated the support (n=1), appreciated the information (n=1), liked the structure (n=1), and liked talking about exercise that suits them (n=1). However, some had technical problems (n=2) and would have preferred to use a phone (n=1).

### Intervention Adherence and Retention

Retention did not differ between intervention groups (χ^2^_1_= 4.7, *P*=.11). Participants who completed at least 1 coaching session had a higher percentage of week 9 survey completers (n=8/11, 73%), compared with other intervention participants (n=50/95, 53%), but this difference was not significant (χ^2^_1_= 1.6, *P*=.21). Just under half (n=50/106, 47%) of participants completed at least 3 of the 4 intervention modules. Intervention adherence was similar for the tailoring + video-coaching group and the tailoring-only group (χ^2^_1_= 2.1, *P*=.15); however, significantly more participants who participated in the coaching sessions completed at least 3 of the 4 intervention modules (n=9/11, 82%) compared with other intervention participants (n=41/95, 43%; χ^2^_1_= 6.0, *P*=.01).

### Website Engagement

The average website visits and minutes spent on the website for the intervention groups were 7.53 (SD 7.14) and 87.07 (SD 77.33) minutes, respectively. The average number of website visits was similar for the tailoring + video-coaching group and the tailoring-only group (*t*_1,103_= 0.05, *P*=.96). Average minutes spent on the website was higher for the tailoring + video-coaching group (mean 99.58 minutes, SD 95.71) than the tailoring-only group (mean 75.25 minutes, SD 52.90), but this was not significantly different (*t*_1103_= 1.60, *P*=.11). Participants who completed the coaching sessions spent a significantly longer time (*t*_1103_= 2.73, *P*=.02) on the intervention website (mean 174.64 minutes, SD 110.11) compared with other intervention participants (mean 77.84 minutes, SD 67.48). Participants who completed the coaching sessions also visited the website more frequently (mean 10.20, SD 3.85) compared with other intervention participants (mean 7.25, SD= 7.36), but this difference was not significant (*t*_1103_= 1.24, *P*=.22).

### Satisfaction

More than two-thirds of the participants were satisfied with the overall program (n=36/53, 68%), while the majority of participants were satisfied with the website usability (n=40/53, 77%), the computer-tailored advice (n=38/53, 76%), and the module questions (n=48/53, 91%). There was no difference between intervention groups on program satisfaction scores (χ^2^_1_= 0.14, *P*=.71). A higher percentage of those who participated in the coaching sessions (n=7/8, 88%) were satisfied with the program compared with other participants (n=29/45, 64%); however, this difference was not statistically significant (χ^2^= 1.66, *P*=.20).

In response to open-ended questions on the overall program, participants mentioned that they liked the convenience (n=4), ease of use (n=4), the information (n=5), emails (n=2), found it motivating (n=5), and liked the accountability (n=2). Participants also mentioned that they would like more contact with a real person (n=7) and thought there were too many questions (n=2). In response to the questions on the advice, participants mentioned that it was easy to understand (n=12), was concise (n=4), laid out well (n=4), was nonjudgmental (n=2), and liked the personalization (n=2). However, some participants thought it was not personalized enough (n=11), did not like the Web-based format (n=4), and learned nothing new (n=2). In response to open-ended questions on the website, some participants mentioned that the website was easy to use (n=14), whereas others thought it was hard to use (n=5). Lastly, some participants thought the website could use more visuals and interesting links (n=6).

### Physical Activity

**Figure 2:**
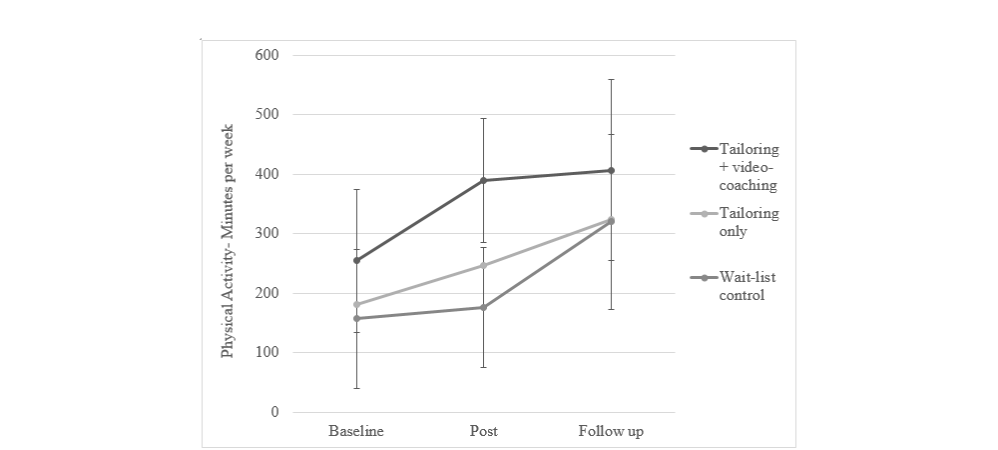
Mean physical activity at baseline, week 9 (post intervention), and 6 months (follow-up), unadjusted. Baseline n=151, postintervention n=83, and follow-up n=59. Note: 95% confidence intervals presented for the coaching and control groups only.

Physical activity (minutes/week) improved from baseline to postintervention (week 9) and from baseline to follow-up (6 months) in all groups (Table 2,Figure 2). According to the linear mixed models analysis, the increase in physical activity from baseline to postintervention in the tailoring + video-coaching group in comparison with the control group was significant (Table 2). No significant difference was found between the intervention groups (Table 2). No significant differences were found between groups on physical activity changes from baseline to follow-up at 6 months.

### Quality of Life

Physical health scores remained relatively constant across each time point (Table 2). Mental health scores remained relatively constant in the control and tailoring + video-coaching group; however, mental health scores dropped in the tailoring-only participants at postintervention (Table 2).

As such, the linear mixed models analysis indicated a significant difference in mental health between both intervention groups from baseline to postintervention (Table 2).

**Table 2:** Physical activity, mental health, and physical health changes by group: results of intention-to-treat analysis using linear mixed models

Outcome variables		Baseline to postintervention			Baseline to follow-up		
		Estimate^a^	*P* value	Cohen’s *d*	Estimate^a^	*P* value	Cohen’s *d*
Physical activity (n=151)							
	Tailoring + video-coaching versus control	140.94 (−254.01 to −27.87)	.01	0.55	66.16 (−244.55 to 112.24)	.46	0.19
	Tailoring + video-coaching versus tailoring-only	35.39 (−148.50 to 77.71)	.54	0.11	−25.16 (−211.74 to 161.43)	.79	0.18
Mental health (n=148)							
	Tailoring + video-coaching versus control	0.04 (−5.87 to 5.94)	.99	0.22	0.36 (−5.65 to6.38)	.90	0.22
	Tailoring + video-coaching versus tailoring-only	−13.18 (−19.10 to −7.26)	.00	0.91	1.43 (−4.91 to 7.77)	.65	0.34
Physical health (n=146)							
	Tailoring + video-coaching versus control	−2.97 (−7.16 to 1.22)	.16	0.41	−2.07 (−6.34 to 2.20)	.34	0.43
	Tailoring + video-coaching versus tailoring-only	−2.58 (−7.00 to 1.83)	.25	0.33	0.16 (−4.32 to 4.65)	.94	0.03

^a^Estimate of tailoring and video-coaching group’s change in the dependent variable in comparison with control and tailoring-only. Higher scores represent greater improvements than the comparison group.

## Discussion

### Feasibility

The first aim of this study was to determine the feasibility of Web-based video-coaching sessions. The low participation and high satisfaction with the coaching sessions suggests that the majority of people participating in Web-based physical activity interventions are reluctant to talk to a coach using a video-calling program, but those who do find it worthwhile. The low participation in the coaching sessions could be explained by the high percentage of coaching participants (37%) who do not use video-calling software. This may be due to the older age of the participants who are less comfortable with technology [4]. Phone calls may have also been received better in this sample. The lower intervention adherence in the video-coaching + tailoring group also suggests that increasing participant burden through an additional video-coaching component may reduce feasibility. The high satisfaction of the video-coaching sessions adds to past research findings of high satisfaction of coaching sessions conducted over the phone [44]. An important reason for the high satisfaction could be the accountability provided through the coaching sessions, as this was the most frequently given positive feedback for the sessions.

The two most common negative comments about the overall program were the lack of personal contact and that it was not personalized enough. Therefore, it is not surprising that those participants who participated in coaching sessions reported higher levels of program satisfaction. Participant satisfaction leads to improved engagement, which is important for intervention effectiveness [45]. In support of this, the participants who completed the coaching sessions were not only more satisfied with the program, but had significantly higher retention, higher adherence, and spent a significantly longer time on the website. This is in line with past research, demonstrating that personal contact in a Web-based intervention improves engagement [46,47]. However, it is possible that the coaching sessions are only effective in some people, and that the coaching sessions in this study may have retained a subsample of participants who were frequent Web users and therefore familiar with video-calling software or were more motivated to begin with. Due to the low participation in the coaching sessions, there were no significant differences in overall retention, adherence, engagement, and satisfaction between the 2 intervention groups. Web-based interventions with computer-tailored advice and coaching sessions may increase retention, adherence, engagement, and satisfaction, but only if they can convince participants to participate in the coaching sessions.

### Effectiveness

The second aim of this study was to assess the effectiveness of the coaching sessions, by comparing physical activity and quality of life changes of the tailoring + video-coaching group with the tailoring-only and control groups. After the 8-week intervention period there was a significant treatment effect of the tailoring + video-coaching physical activity intervention on physical activity compared with no intervention. The improvement in physical activity compared with the control group (116 minutes per week, unadjusted) resulted in a moderate effect size and is considered clinically significant because of the large health effects seen from doing even small amounts of physical activity [48]. This finding is in line with findings that activity counseling over the phone [49], via email [50], and computer-tailored advice [13] improve participants’ physical activity in comparison with a control group. Few studies have specifically tested the effectiveness of counseling through video calls in physical activity interventions. Pilutti et al [51] found that video-coaching to promote physical activity in patients with multiple sclerosis was effective. However, the coaching group’s significant improvement in physical activity compared with the control group, in our study, could be due to the computer-tailored advice.

The tailoring + video-coaching group participants improved their physical activity 27 minutes per week (unadjusted) more in comparison with the tailoring-only group; however, no significant between-group differences were found. The availability of human support may have improved the overall physical activity of the tailoring + video-coaching group. Participants who needed to discuss their computer-tailored advice were able to. Limited studies have compared the effectiveness of coaching in addition to computer-tailored advice. Van Hoye et al [52] compared physical activity self-monitoring with and without additional face-to-face coaching sessions. They found that the coaching group had significantly greater physical activity improvements than the self-monitoring only group. An earlier study tested the effectiveness of email coaching in addition to a basic Web-based weight loss intervention [53]. The email-coaching group had significantly greater weight loss outcomes than the Web-based intervention only group. However, the effectiveness of coaching found in these studies may be due to the minimal nature of the comparison interventions (generic physical activity information + self-monitoring). The physical activity advice given in our study was highly tailored to participants’ physical activity behavior, demographics, and psychosocial correlates of physical activity. This feedback might be enough to optimize physical activity outcomes (as demonstrated by a 172 minutes/week increase in physical activity in this group). The lack of significant differences between the intervention groups could also be due to low adherence to the coaching sessions, which may have reduced the effectiveness of the intervention in the tailoring + video-coaching group. The detailed computer-tailored advice may have discouraged participants to adhere to their coaching sessions as they were satisfied with the computer-tailored advice. Core intervention content may need to be delivered in the coaching sessions to promote higher adherence. Therefore, more research is needed to determine whether Web-based coaching is more effective than stand-alone computer-tailored advice.

The physical activity levels in the tailoring + video-coaching and tailoring-only groups were maintained at 6 months. There were, however, no significant between-group differences in physical activity changes from baseline to 6 months. This was due to the control group participants increasing their activity from 9 weeks to 6 months after the intervention. The absence of between-group physical activity changes at 6 months after the intervention is not uncommon in physical activity interventions; however, it is usually due to the intervention group’s decline in physical activity rather than a physical activity increase in the control group [54].

There were no differences the physical health component of quality of life over time for any of the groups. This may be due to the sample not being large enough to detect subtle improvements or the overall high level of physical activity and physical health in the sample at baseline. Although previous studies have established a positive association between physical activity and quality of life [55], the effect of increased physical activity on quality of life is mainly seen in clinical samples that have a lower quality of life at baseline [56]. The significant reduction in quality of life in relation to mental health in the tailoring-only group from baseline to postintervention in comparison was unexpected, as this group’s participants did increase their physical activity at a clinically significant level and previous studies have associated this with an increase in quality of life [55].

### Limitations

Limitations of the research include self-reported physical activity, which may be subject to social desirability bias. Participants and researchers were not blinded to group assignment, which may have biased results. The sample was predominantly female, white, and educated. Therefore, the results may not be generalizable to males, other cultures, and low socioeconomic groups. A high percentage of the sample was not in paid employment when compared with the Australian population data (43% vs 33%) [57]. This may be due to the high percentage of females and the older age of the participants. It could also be due to the people not in paid employment having more time to participate. Therefore, the results may not be generalizable to people in paid employment.

Furthermore, 40% of the participants were physically active at baseline despite the target group being inactive adults. This is not uncommon in physical activity studies [58], although results may not be generalizable to physically inactive Australians who are most in need of increasing their activity. The sample may have a high level of computer and Internet literacy as the majority of participants were reached through Facebook. Therefore, the findings may not be fully generalizable to people with a lower computer and Internet literacy. The additional phone calls the coaching participants received encouraging them to complete the coaching calls may have affected their physical activity levels. However, this is expected to be minimal due to the phone discussion being focused on scheduling the coaching call rather than their physical activity. Objective measurement of physical activity (eg, accelerometry) is needed to confirm the findings of this study. Although we were able to detect a difference in physical activity changes between the tailoring + video-coaching and tailoring-only groups, the analysis was underpowered to detect other differences between the groups as the required sample size of 100 per group was not met. The low retention is also a significant limitation. Low retention is common in Web-based programs, potentially due to the minimal face-to-face contact through either recruitment or the intervention. Similar retention rates have been observed in other Web-based health behavior interventions [59,60]. Retention is likely to be even lower outside of a randomized controlled trial setting that included reminder calls from researchers.

### Conclusions

Combined Web-based video-coaching and computer-tailored advice was effective in comparison with a control group; however, only small nonsignificant improvements were seen when video-coaching was included in addition to computer-tailored advice in a Web-based physical activity intervention. Only a small percentage of participants adhered to Web-based video-coaching sessions, but those who participated were highly satisfied and more engaged in the intervention. Further research should investigate how adherence to Web-based coaching sessions can be improved.

## Appendix

Multimedia Appendix 1 CONSORT-EHEALTH checklist V1.6.2 [29].

Multimedia Appendix 2 Intervention landing page.

Multimedia Appendix 3 Participant information sheet.

Multimedia Appendix 4 Tailored advice with graph.

Multimedia Appendix 5 Text-only tailored advice.

Multimedia Appendix 6 Action plan output.

Multimedia Appendix 7 Satisfaction survey.

## Abbreviations

BMI: body mass index
