# Supplementary material for: Web-Based Video-Coaching to Assist an Automated Computer-Tailored Physical Activity Intervention for Inactive Adults: A Randomized Controlled Trial
Source: J Med Internet Res. 2016 Aug 12;18(8):e223. doi: 10.2196/jmir.5664 (PMC5002066; doi:10.2196/jmir.5664)
Supplement: Multimedia Appendix 1 [file jmir_v18i8e223_app1.pdf]

CONSORT-EHEALTH (V 1.6.1) - Submission/Publication Form

The CONSORT-EHEALTH checklist is intended for authors of randomized trials evaluating web-based and Internet-based applications/interventions, including mobile interventions, electronic games (incl multiplayer games), social media, certain telehealth applications, and other interactive and/or networked electronic applications. Some of the items (e.g. all subitems under item 5 - description of the intervention) may also be applicable for other study designs.

The goal of the CONSORT EHEALTH checklist and guideline is to be
a) a guide for reporting for authors of RCTs,
b) to form a basis for appraisal of an ehealth trial (in terms of validity)

CONSORT-EHEALTH items/subitems are MANDATORY reporting items for studies published in the Journal of Medical Internet Research and other journals / scientific societies endorsing the checklist.

Items numbered 1., 2., 3., 4a., 4b etc are original CONSORT or CONSORT-NPT (non-pharmacologic treatment) items.
Items with Roman numerals (i., ii, iii, iv etc.) are CONSORT-EHEALTH extensions/clarifications.

As the CONSORT-EHEALTH checklist is still considered in a formative stage, we would ask that you also RATE ON A SCALE OF 1-5 how important/useful you feel each item is FOR THE PURPOSE OF THE CHECKLIST and reporting guideline (optional).

Mandatory reporting items are marked with a red *.
In the textboxes, either copy & paste the relevant sections from your manuscript into this form - please include any quotes from your manuscript in QUOTATION MARKS,
or answer directly by providing additional information not in the manuscript, or elaborating on why the item was not relevant for this study.

YOUR ANSWERS WILL BE PUBLISHED AS A SUPPLEMENTARY FILE TO YOUR PUBLICATION IN JMIR AND ARE CONSIDERED PART OF YOUR PUBLICATION (IF ACCEPTED).
Please fill in these questions diligently. Information will not be copyedited, so please use proper spelling and grammar, use correct capitalization, and avoid abbreviations.

DO NOT FORGET TO SAVE AS PDF _AND_ CLICK THE SUBMIT BUTTON SO YOUR ANSWERS ARE IN OUR DATABASE !!!

Citation Suggestion (if you append the pdf as Appendix we suggest to cite this paper in the caption):
Eysenbach G, CONSORT-EHEALTH Group
CONSORT-EHEALTH: Improving and Standardizing Evaluation Reports of Web-based and Mobile Health Interventions
J Med Internet Res 2011;13(4):e126
URL: http://www.jmir.org/2011/4/e126/
doi: 10.2196/jmir.1923
PMID: 22209829

* Required

Your name *
First Last

Primary Affiliation (short), City, Country *

University of Toronto, Toronto, Canada

Your e-mail address *

abc@gmail.com

Title of your manuscript *

Provide the (draft) title of your manuscript.

Web-based video-coaching to assist an automated computer-tailored physical activity intervention for inactive adults: A randomised controlled trial

Article Preparation Status/Stage *

At which stage in your article preparation are you currently (at the time you fill in this form)

- ○ not submitted yet - in early draft status
- ○ not submitted yet - in late draft status, just before submission
- ○ submitted to a journal but not reviewed yet
- ○ submitted to a journal and after receiving initial reviewer comments
- ○ submitted to a journal and accepted, but not published yet
- ○ published
- ○ Other:

Journal *

If you already know where you will submit this paper (or if it is already submitted), please provide the journal name (if it is not JMIR, provide the journal name under "other")

- ○ not submitted yet / unclear where I will submit this
- ○ Journal of Medical Internet Research (JMIR)
- ○ Other:

Manuscript tracking number *

If this is a JMIR submission, please provide the manuscript tracking number under "other" (The ms tracking number can be found in the submission acknowledgement email, or when you login as author in JMIR. If the paper is already published in JMIR, then the ms tracking number is the four-digit number at the end of the DOI, to be found at the bottom of each published article in JMIR)

- ○ no ms number (yet) / not (yet) submitted to / published in JMIR
- ○ Other:

TITLE AND ABSTRACT

1a) TITLE: Identification as a randomized trial in the title

1a) Does your paper address CONSORT item 1a? *

I.e does the title contain the phrase "Randomized Controlled Trial"? (if not, explain the reason under "other")

- ○ yes
- ○ Other: [ ]

1a-i) Identify the mode of delivery in the title

Identify the mode of delivery. Preferably use “web-based” and/or “mobile” and/or “electronic game” in the title. Avoid ambiguous terms like “online”, “virtual”, “interactive”. Use “Internet-based” only if Intervention includes non-web-based Internet components (e.g. email), use “computer-based” or “electronic” only if offline products are used. Use “virtual” only in the context of “virtual reality” (3-D worlds). Use “online” only in the context of “online support groups”. Complement or substitute product names with broader terms for the class of products (such as “mobile” or “smart phone” instead of “iphone”), especially if the application runs on different platforms.

	1	2	3	4	5	
subitem not at all important	○	○	○	○	○	essential

Does your paper address subitem 1a-i? *

Copy and paste relevant sections from manuscript title (include quotes in quotation marks "like this" to indicate direct quotes from your manuscript), or elaborate on this item by providing additional information not in the ms, or briefly explain why the item is not applicable/relevant for your study

"Web-based." "A randomised controlled trial."

1a-ii) Non-web-based components or important co-interventions in title

Mention non-web-based components or important co-interventions in title, if any (e.g., “with telephone support”).

	1	2	3	4	5	
subitem not at all important	○	○	○	○	○	essential

Does your paper address subitem 1a-ii?

Copy and paste relevant sections from manuscript title (include quotes in quotation marks "like this" to indicate direct quotes from your manuscript), or elaborate on this item by providing additional information not in the ms, or briefly explain why the item is not applicable/relevant for your study

There are no non-web-based components or co-interventions included in the intervention.

1a-iii) Primary condition or target group in the title

Mention primary condition or target group in the title, if any (e.g., "for children with Type I Diabetes") Example: A Web-based and Mobile Intervention with Telephone Support for Children with Type I Diabetes: Randomized Controlled Trial

	1	2	3	4	5	
subitem not at all important	○	○	○	○	○	essential

Does your paper address subitem 1a-iii? *

Copy and paste relevant sections from manuscript title (include quotes in quotation marks "like this" to indicate direct quotes from your manuscript), or elaborate on this item by providing additional information not in the ms, or briefly explain why the item is not applicable/relevant for your study

"Intervention for inactive adults."

1b) ABSTRACT: Structured summary of trial design, methods, results, and conclusions

NPT extension: Description of experimental treatment, comparator, care providers, centers, and blinding status.

1b-i) Key features/functionalities/components of the intervention and comparator in the METHODS section of the ABSTRACT

Mention key features/functionalities/components of the intervention and comparator in the abstract. If possible, also mention theories and principles used for designing the site. Keep in mind the needs of systematic reviewers and indexers by including important synonyms. (Note: Only report in the abstract what the main paper is reporting. If this information is missing from the main body of text, consider adding it)

	1	2	3	4	5	
subitem not at all important	○	○	○	○	○	essential

Does your paper address subitem 1b-i? *

Copy and paste relevant sections from the manuscript abstract (include quotes in quotation marks "like this" to indicate direct quotes from your manuscript), or elaborate on this item by providing additional information not in the ms, or briefly explain why the item is not applicable/relevant for your study

"Participants were assigned to one of three groups: 1) tailoring + video-coaching where participants received an 8 week computer-tailored web-based physical activity intervention (‘My Activity Coach’) including four 10 minute coaching sessions with a behavioural expert using a web-based video-calling program (e.g. Skype) (n=52); 2) tailoring-only where participants received the same intervention without the coaching sessions (n=54); and 3) a wait-list control group (n=45)."

1b-ii) Level of human involvement in the METHODS section of the ABSTRACT

Clarify the level of human involvement in the abstract, e.g., use phrases like “fully automated” vs. “therapist/nurse/care provider/physician-assisted” (mention number and expertise of providers involved, if any). (Note: Only report in the abstract what the main paper is reporting. If this information is missing from the main body of text, consider adding it)

	1	2	3	4	5	
subitem not at all important	○	○	○	○	○	essential

Does your paper address subitem 1b-ii?

Copy and paste relevant sections from the manuscript abstract (include quotes in quotation marks "like this" to indicate direct quotes from your manuscript), or elaborate on this item by providing additional information not in the ms, or briefly explain why the item is not applicable/relevant for your study

"The purpose of this study was to determine the feasibility and effectiveness of brief video-based coaching in addition to fully automated computer-tailored advice in a web-based physical activity intervention."

1b-iii) Open vs. closed, web-based (self-assessment) vs. face-to-face assessments in the METHODS section of the ABSTRACT

Mention how participants were recruited (online vs. offline), e.g., from an open access website or from a clinic or a closed online user group (closed usergroup trial), and clarify if this was a purely web-based trial, or there were face-to-face components (as part of the intervention or for assessment). Clearly say if outcomes were self-assessed through questionnaires (as common in web-based trials). Note: In traditional offline trials, an open trial (open-label trial) is a type of clinical trial in which both the researchers and participants know which treatment is being administered. To avoid confusion, use “blinded” or “unblinded” to indicated the level of blinding instead of “open”, as “open” in web-based trials usually refers to “open access” (i.e. participants can self-enrol). (Note: Only report in the abstract what the main paper is reporting. If this information is missing from the main body of text, consider adding it)

	1	2	3	4	5	
subitem not at all important	○	○	○	○	○	essential

Does your paper address subitem 1b-iii?

Copy and paste relevant sections from the manuscript abstract (include quotes in quotation marks "like this" to indicate direct quotes from your manuscript), or elaborate on this item by providing additional information not in the ms, or briefly explain why the item is not applicable/relevant for your study

"Demographics were measured at baseline, intervention satisfaction at week 9 and physical activity at baseline, week 9 and 6 months by web-based self-report surveys."

1b-iv) RESULTS section in abstract must contain use data

Report number of participants enrolled/assessed in each group, the use/uptake of the intervention (e.g., attrition/adherence metrics, use over time, number of logins etc.), in addition to primary/secondary outcomes. (Note: Only report in the abstract what the main paper is reporting. If this information is missing from the main body of text, consider adding it)

	1	2	3	4	5	
subitem not at all important	○	○	○	○	○	essential

Does your paper address subitem 1b-iv?

Copy and paste relevant sections from the manuscript abstract (include quotes in quotation marks "like this" to indicate direct quotes from your manuscript), or elaborate on this item by providing additional information not in the ms, or briefly explain why the item is not applicable/relevant for your study

"A total of 23 tailoring + video coaching participants, 30 tailoring-only participants and 30 control participants completed the post intervention survey (55% retention)."

"Improved intervention adherence (82% completed 3 or 4 modules vs 43%, P=.01) and engagement (110 minutes spent on the website vs 78 minutes, P=.02) compared to other participants."

1b-v) CONCLUSIONS/DISCUSSION in abstract for negative trials

Conclusions/Discussions in abstract for negative trials: Discuss the primary outcome - if the trial is negative (primary outcome not changed), and the intervention was not used, discuss whether negative results are attributable to lack of uptake and discuss reasons. (Note: Only report in the abstract what the main paper is reporting. If this information is missing from the main body of text, consider adding it)

	1	2	3	4	5	
subitem not at all important	○	○	○	○	○	essential

Does your paper address subitem 1b-v?

Copy and paste relevant sections from the manuscript abstract (include quotes in quotation marks "like this" to indicate direct quotes from your manuscript), or elaborate on this item by providing additional information not in the ms, or briefly explain why the item is not applicable/relevant for your study

Due to the low retention and adherence (specified in results) we concluded that more research is needed to determine whether online coaching is more effective than stand-alone computer-tailored advice:

"The findings support the effectiveness of providing online video-coaching and computer-tailored advice in a web-based physical activity intervention. More research is needed to determine whether online coaching is more effective than stand-alone computer-tailored advice."

INTRODUCTION

2a) In INTRODUCTION: Scientific background and explanation of rationale

2a-i) Problem and the type of system/solution

Describe the problem and the type of system/solution that is object of the study: intended as stand-alone intervention vs. incorporated in broader health care program? Intended for a particular patient population? Goals of the intervention, e.g., being more cost-effective to other interventions, replace or complement other solutions? (Note: Details about the intervention are provided in “Methods” under 5)

	1	2	3	4	5	
subitem not at all important	○	○	○	○	○	essential

Does your paper address subitem 2a-i? *

Copy and paste relevant sections from the manuscript (include quotes in quotation marks "like this" to indicate direct quotes from your manuscript), or elaborate on this item by providing additional information not in the ms, or briefly explain why the item is not applicable/relevant for your study

The problem:
"The effectiveness of web-based physical activity interventions is well established, however problems with low engagement and high drop-out rates limit their effectiveness to achieve long-term behaviour changes."

Type of intervention and population:
"The first aim of the study is to determine the feasibility of brief video-coaching, when used to discuss previously received computer-tailored physical activity advice, in a stand-alone web-based intervention for inactive adults."

2a-ii) Scientific background, rationale: What is known about the (type of) system

Scientific background, rationale: What is known about the (type of) system that is the object of the study (be sure to discuss the use of similar systems for other conditions/diagnoses, if appropiate), motivation for the study, i.e. what are the reasons for and what is the context for this specific study, from which stakeholder viewpoint is the study performed, potential impact of findings [2]. Briefly justify the choice of the comparator.

	1	2	3	4	5	
subitem not at all important	○	○	○	○	○	essential

Does your paper address subitem 2a-ii? *

Copy and paste relevant sections from the manuscript (include quotes in quotation marks "like this" to indicate direct quotes from your manuscript), or elaborate on this item by providing additional information not in the ms, or briefly

explain why the item is not applicable/relevant for your study

"Personalised health advice through online coaching sessions or computer-tailored feedback engages participants and improves the effectiveness of web-based health behaviour interventions. Both online coaching sessions and computer-tailored advice provide support similar to traditional face-to-face coaching sessions, at a lower cost with fewer geographical restrictions."

"To our knowledge no health behaviour interventions have combined computer-tailored advice with online video-coaching. This approach may improve intervention outcomes by utilising the benefits of both methods. Providing computer-tailored advice can limit the time required from a video-coach, therefore limiting costs, as well as reducing reliance on the knowledge and expertise of the coach. A brief coaching-session can add an element of social support, as well as further explanation, personalisation and interpretation of theory-based computer-tailored advice received by participants at an earlier time."

2b) In INTRODUCTION: Specific objectives or hypotheses

Does your paper address CONSORT subitem 2b? *

Copy and paste relevant sections from the manuscript (include quotes in quotation marks "like this" to indicate direct quotes from your manuscript), or elaborate on this item by providing additional information not in the ms, or briefly explain why the item is not applicable/relevant for your study

"The first aim of the study is to determine the feasibility of brief video-coaching, when used to discuss previously received computer-tailored physical activity advice, in a stand-alone web-based intervention for inactive adults. Feasibility will be determined by adherence and satisfaction of the coaching sessions and comparing intervention retention, adherence, website engagement and satisfaction of the tailoring + video-coaching and tailoring only groups. The second aim is to test the effectiveness of the video-coaching sessions in terms of physical activity and quality of life outcomes. It was hypothesised that computer-tailoring in combination with video-coaching would result in greater retention, adherence, engagement and satisfaction with the intervention, compared to a computer-tailored only group and a wait-list control group. It was also hypothesised that computer-tailoring and video-coaching would result in greater improvements in quality of life and physical activity compared to a computer-tailored only group and a wait-list control group."

METHODS

3a) Description of trial design (such as parallel, factorial) including allocation ratio

Does your paper address CONSORT subitem 3a? *

Copy and paste relevant sections from the manuscript (include quotes in quotation marks "like this" to indicate direct quotes from your manuscript), or elaborate on this item by providing additional information not in the ms, or briefly explain why the item is not applicable/relevant for your study

"Eligible participants were randomly assigned based on a sequence (not concealed) of random numbers between 1 and 3 to one of three study arms: tailoring + video-coaching, tailoring-only or wait-list control."

3b) Important changes to methods after trial commencement (such as eligibility criteria), with reasons

Does your paper address CONSORT subitem 3b? *

Copy and paste relevant sections from the manuscript (include quotes in quotation marks "like this" to indicate direct

quotes from your manuscript), or elaborate on this item by providing additional information not in the ms, or briefly explain why the item is not applicable/relevant for your study

"Due to many individuals beginning and failing to complete the screening questionnaire, conducting the screening questionnaire by phone was trialled (after receiving an ethics amendment from the Central Queensland University's Human Ethics Committee). This was done for 15 prospective participants and discontinued due to failure to increase screening completions."

3b-i) Bug fixes, Downtimes, Content Changes

Bug fixes, Downtimes, Content Changes: ehealth systems are often dynamic systems. A description of changes to methods therefore also includes important changes made on the intervention or comparator during the trial (e.g., major bug fixes or changes in the functionality or content) (5-iii) and other "unexpected events" that may have influenced study design such as staff changes, system failures/downtimes, etc. [2].

	1	2	3	4	5	
subitem not at all important	○	○	○	○	○	essential

Does your paper address subitem 3b-i?

Copy and paste relevant sections from the manuscript (include quotes in quotation marks "like this" to indicate direct quotes from your manuscript), or elaborate on this item by providing additional information not in the ms, or briefly explain why the item is not applicable/relevant for your study

"The web-based booking system for coaching participants was changed halfway through the trial as the first booking system was discontinued."

4a) Eligibility criteria for participants

Does your paper address CONSORT subitem 4a? *

Copy and paste relevant sections from the manuscript (include quotes in quotation marks "like this" to indicate direct quotes from your manuscript), or elaborate on this item by providing additional information not in the ms, or briefly explain why the item is not applicable/relevant for your study

"People were eligible to participate if they were English speaking Australian adults (over 18 years). Participants were excluded if they were pregnant, at risk of injury or ill health from increasing their physical activity (as assessed by the Physical Activity Readiness Questionnaire), or if they were already meeting the physical activity recommendations (as assessed via a single item asking if participants participated in 30 minutes of physical activity on most days)."

4a-i) Computer / Internet literacy

Computer / Internet literacy is often an implicit "de facto" eligibility criterion - this should be explicitly clarified.

	1	2	3	4	5	
subitem not at all important	○	○	○	○	○	essential

Does your paper address subitem 4a-i?

Copy and paste relevant sections from the manuscript (include quotes in quotation marks "like this" to indicate direct quotes from your manuscript), or elaborate on this item by providing additional information not in the ms, or briefly explain why the item is not applicable/relevant for your study

"It is likely that the intervention attracted participants with a high internet literacy."

4a-ii) Open vs. closed, web-based vs. face-to-face assessments:

Open vs. closed, web-based vs. face-to-face assessments: Mention how participants were recruited (online vs. offline), e.g., from an open access website or from a clinic, and clarify if this was a purely web-based trial, or there were face-to-face components (as part of the intervention or for assessment), i.e., to what degree got the study team to know the participant. In online-only trials, clarify if participants were quasi-anonymous and whether having multiple identities was possible or whether technical or logistical measures (e.g., cookies, email confirmation, phone calls) were used to detect/prevent these.

	1	2	3	4	5	
subitem not at all important	○	○	○	○	○	essential

Does your paper address subitem 4a-ii? *

Copy and paste relevant sections from the manuscript (include quotes in quotation marks "like this" to indicate direct quotes from your manuscript), or elaborate on this item by providing additional information not in the ms, or briefly explain why the item is not applicable/relevant for your study

Recruitment:
"Print and Internet advertising was used to recruit participants from a number of Australian metropolitan and regional cities (Sydney, Melbourne, Perth, Brisbane, Rockhampton, Bundaberg, Mackay and Townsville). Print advertising included newspaper advertisements and articles, posters and leaflets displayed in health clinics and leaflets distributed to peoples' homes. The Internet advertising included links displayed on community websites and paid advertisements on Google and Facebook."

Participant contacts:
"Participants in the tailoring-only group received an email reminding them of the tailored advice they received in the previous module to ensure both intervention groups received the same number of contacts."

"Participants received up to four reminder emails and a reminder phone call when they didn't complete the survey required for each module."

4a-iii) Information giving during recruitment

Information given during recruitment. Specify how participants were briefed for recruitment and in the informed consent procedures (e.g., publish the informed consent documentation as appendix, see also item X26), as this information may have an effect on user self-selection, user expectation and may also bias results.

	1	2	3	4	5	
subitem not at all important	○	○	○	○	○	essential

Does your paper address subitem 4a-iii?

Copy and paste relevant sections from the manuscript (include quotes in quotation marks "like this" to indicate direct quotes from your manuscript), or elaborate on this item by providing additional information not in the ms, or briefly explain why the item is not applicable/relevant for your study

"Information about the study including the affiliation with Central Queensland University was available on the landing page of the intervention website."
"The consent form and then baseline questionnaire were administered through the intervention website for all groups."

The informed consent form will be published as an appendix.

4b) Settings and locations where the data were collected

Does your paper address CONSORT subitem 4b? *

Copy and paste relevant sections from the manuscript (include quotes in quotation marks "like this" to indicate direct quotes from your manuscript), or elaborate on this item by providing additional information not in the ms, or briefly explain why the item is not applicable/relevant for your study

"Questionnaires were administered through the intervention website."
"Completion of the coaching sessions, the length of the coaching sessions and reasons for missed coaching sessions were recorded by the coach. Intervention retention and adherence were assessed by recording participants' completion of the research surveys and intervention modules respectively, and website engagement was measured through Google Analytics."

4b-i) Report if outcomes were (self-)assessed through online questionnaires

Clearly report if outcomes were (self-)assessed through online questionnaires (as common in web-based trials) or otherwise.

	1	2	3	4	5	
subitem not at all important	○	○	○	○	○	essential

Does your paper address subitem 4b-i? *

Copy and paste relevant sections from the manuscript (include quotes in quotation marks "like this" to indicate direct quotes from your manuscript), or elaborate on this item by providing additional information not in the ms, or briefly explain why the item is not applicable/relevant for your study

"Questionnaires were administered through the intervention website."

4b-ii) Report how institutional affiliations are displayed

Report how institutional affiliations are displayed to potential participants [on ehealth media], as affiliations with prestigious hospitals or universities may affect volunteer rates, use, and reactions with regards to an intervention.(Not a required item – describe only if this may bias results)

	1	2	3	4	5	
subitem not at all important	○	○	○	○	○	essential

Does your paper address subitem 4b-ii?

Copy and paste relevant sections from the manuscript (include quotes in quotation marks "like this" to indicate direct quotes from your manuscript), or elaborate on this item by providing additional information not in the ms, or briefly explain why the item is not applicable/relevant for your study

"Information about the study including the affiliation with Central Queensland University was available on the landing page of the intervention website."

5) The interventions for each group with sufficient details to allow replication, including how and when they were actually administered

5-i) Mention names, credential, affiliations of the developers, sponsors, and owners

Mention names, credential, affiliations of the developers, sponsors, and owners [6] (if authors/evaluators are owners or developer of the software, this needs to be declared in a “Conflict of interest” section or mentioned elsewhere in the manuscript).

	1	2	3	4	5	
subitem not at all important	○	○	○	○	○	essential

Does your paper address subitem 5-i?

Copy and paste relevant sections from the manuscript (include quotes in quotation marks "like this" to indicate direct quotes from your manuscript), or elaborate on this item by providing additional information not in the ms, or briefly explain why the item is not applicable/relevant for your study

"Conflicts of Interest
CV owns the computer-tailoring software which was used in the web-based intervention. The authors declare that there are no other conflicts of Interest."
"Updates were conducted by a website developer who also created the original intervention website."

5-ii) Describe the history/development process

Describe the history/development process of the application and previous formative evaluations (e.g., focus groups, usability testing), as these will have an impact on adoption/use rates and help with interpreting results.

	1	2	3	4	5	
subitem not at all important	○	○	○	○	○	essential

Does your paper address subitem 5-ii?

Copy and paste relevant sections from the manuscript (include quotes in quotation marks "like this" to indicate direct quotes from your manuscript), or elaborate on this item by providing additional information not in the ms, or briefly explain why the item is not applicable/relevant for your study

"The modules and intervention website were adapted from an earlier 2-module web-based intervention with computer-tailored advice for inactive adults. Focus groups were conducted to inform development of this prior intervention."

5-iii) Revisions and updating

Revisions and updating. Clearly mention the date and/or version number of the application/intervention (and comparator, if applicable) evaluated, or describe whether the intervention underwent major changes during the evaluation process, or whether the development and/or content was "frozen" during the trial. Describe dynamic components such as news feeds or changing content which may have an impact on the replicability of the intervention (for unexpected events see item 3b).

	1	2	3	4	5	
subitem not at all important	○	○	○	○	○	essential

Does your paper address subitem 5-iii?

Copy and paste relevant sections from the manuscript (include quotes in quotation marks "like this" to indicate direct quotes from your manuscript), or elaborate on this item by providing additional information not in the ms, or briefly explain why the item is not applicable/relevant for your study

No revisions to the intervention were conducted during the intervention trial. The content participants could access did however change throughout the trial as they were given access to a new modules and ab action planing tool throughout the intervention:

"The 8-week 'My Activity Coach' web-based intervention delivered a new module of tailored advice to participants every two weeks."

"An action planning tool became available to participants after Module 2."

5-iv) Quality assurance methods

Provide information on quality assurance methods to ensure accuracy and quality of information provided [1], if applicable.

	1	2	3	4	5	
subitem not at all important	○	○	○	○	○	essential

Does your paper address subitem 5-iv?

Copy and paste relevant sections from the manuscript (include quotes in quotation marks "like this" to indicate direct quotes from your manuscript), or elaborate on this item by providing additional information not in the ms, or briefly explain why the item is not applicable/relevant for your study

The first author of the manuscript (SA) managed the intervention development and evaluation. SA has ensured that all information provided on the intervention is correct.

5-v) Ensure replicability by publishing the source code, and/or providing screenshots/screen-capture video, and/or providing flowcharts of the algorithms used

Ensure replicability by publishing the source code, and/or providing screenshots/screen-capture video, and/or providing flowcharts of the algorithms used. Replicability (i.e., other researchers should in principle be able to replicate the study) is a hallmark of scientific reporting.

	1	2	3	4	5	
subitem not at all important	○	○	○	○	○	essential

Does your paper address subitem 5-v?

Copy and paste relevant sections from the manuscript (include quotes in quotation marks "like this" to indicate direct quotes from your manuscript), or elaborate on this item by providing additional information not in the ms, or briefly explain why the item is not applicable/relevant for your study

Details of the content in each intervention module, intervention screen shots and the website address are published in the intervention trial's protocol paper.

5-vi) Digital preservation

Digital preservation: Provide the URL of the application, but as the intervention is likely to change or disappear over the course of the years; also make sure the intervention is archived (Internet Archive, webcitation.org, and/or publishing the source code or screenshots/videos alongside the article). As pages behind login screens cannot be archived, consider creating demo pages which are accessible without login.

	1	2	3	4	5	
subitem not at all important	○	○	○	○	○	essential

Does your paper address subitem 5-vi?

Copy and paste relevant sections from the manuscript (include quotes in quotation marks "like this" to indicate direct quotes from your manuscript), or elaborate on this item by providing additional information not in the ms, or briefly explain why the item is not applicable/relevant for your study

The website has been archived through webcitation.org and referenced in the manuscript.

"The 8-week ‘My Activity Coach’ web-based intervention delivered a new module of tailored advice to participants every two weeks [21]."

5-vii) Access

Access: Describe how participants accessed the application, in what setting/context, if they had to pay (or were paid) or not, whether they had to be a member of specific group. If known, describe how participants obtained “access to the platform and Internet” [1]. To ensure access for editors/reviewers/readers, consider to provide a “backdoor” login account or demo mode for reviewers/readers to explore the application (also important for archiving purposes, see vi).

	1	2	3	4	5	
subitem not at all important	○	○	○	○	○	essential

Does your paper address subitem 5-vii? *

Copy and paste relevant sections from the manuscript (include quotes in quotation marks "like this" to indicate direct quotes from your manuscript), or elaborate on this item by providing additional information not in the ms, or briefly explain why the item is not applicable/relevant for your study

Participants could visit the intervention website in response to the advertising at any time that suited them. The process is explained in the intervention protocol section of the manuscript:

"Information about the study including the affiliation with Central Queensland University was available on the landing page of the intervention website. To assess an individuals’ eligibility, how they heard about the program and collect contact details, a screening questionnaire was delivered through the intervention website. Eligible participants were randomly assigned based on a sequence (not concealed) of random numbers between 1 and 3 to one of three study arms: tailoring + video-coaching, tailoring-only or wait-list control. This was done in blocks of 15 participants. SA generated the random allocation sequence and assigned participants to groups. Participants remained blind to their condition until after completing all baseline measures. Participants began the intervention on the Monday following their recruitment."

Participants were not paid, but were put into the draw to win one of 39 prizes if they completed the research surveys:

"Participants who completed all surveys went in the draw to win one of 30 pedometers, 6 fit bits and 3 heart rate monitors."

5-viii) Mode of delivery, features/functionalities/components of the intervention and comparator, and the theoretical framework

Describe mode of delivery, features/functionalities/components of the intervention and comparator, and the theoretical framework [6] used to design them (instructional strategy [1], behaviour change techniques, persuasive features, etc., see e.g., [7, 8] for terminology). This includes an in-depth description of the content (including where it is coming from and who developed it) [1],” whether [and how] it is tailored to individual circumstances and allows users to track their progress and receive feedback” [6]. This also includes a description of communication delivery channels and – if computer-mediated communication is a component – whether communication was synchronous or asynchronous [6]. It also includes information on presentation strategies [1], including page design principles, average amount of text on pages, presence of hyperlinks to other resources, etc. [1].

	1	2	3	4	5	
subitem not at all important	○	○	○	○	○	essential

Does your paper address subitem 5-viii? *

Copy and paste relevant sections from the manuscript (include quotes in quotation marks "like this" to indicate direct quotes from your manuscript), or elaborate on this item by providing additional information not in the ms, or briefly explain why the item is not applicable/relevant for your study

"The 8-week ‘My Activity Coach’ web-based intervention delivered a new module of tailored advice to participants every two weeks. Each module required participants to complete a brief online questionnaire about their physical activity and psychosocial correlates of physical activity. Feedback was then provided based on their responses to the questionnaire. Participants received up to four reminder emails and a reminder phone call when they didn’t complete the survey required for each module. The tailored advice was based on behaviour change theory [Theory of Planned Behaviour (TPB)] and communication theory [Elaboration Likelihood Model (ELM)]. Each module began with a graph including bars to represent participants’ current physical activity, their physical activity during the previous modules, as well as the minimum and optimal physical activity recommendations. Module 1, titled ‘Are you active enough’, explained the physical activity recommendations and health benefits of physical activity tailored to their BMI, age and level of physical activity. The module ended with a suggested goal (based on their current activity level) to work towards until the next module. Module 2, titled ‘Let’s set some goals’, provided participants with information on goal setting and action planning. Module 3, titled ‘Physical activity and your environment’, delivered tailored information on using participants’ social and physical environments to increase their physical activity. Module 4, titled ‘Staying active’, addressed relapse prevention. Participants also received tailored advice on their perceived benefits and barriers to being active and self-efficacy to become more active throughout the modules."

"An action planning tool became available to participants after Module 2. The tool allowed participants to create an action plan for up to 4 activities (specifying where, when, for how long, and with whom they will be active over the following two weeks). Participants could print a calendar-based overview of their action plan. The coaching group’s 10 to 15 minute bi-weekly video-coaching sessions were conducted through an online video-calling program of participants’ choice (e.g. Skype, Google Hangout, Yahoo Messenger and Face Time). During the session the Activity Coach commented on the tailored advice participants received in the module from the previous week, answered any questions participants had, and provided encouragement, support and accountability."

More information on the intervention can be found in the intervention trial's protocol paper.

5-ix) Describe use parameters

Describe use parameters (e.g., intended “doses” and optimal timing for use). Clarify what instructions or recommendations were given to the user, e.g., regarding timing, frequency, heaviness of use, if any, or was the intervention used ad libitum.

	1	2	3	4	5	
subitem not at all important	○	○	○	○	○	essential

Does your paper address subitem 5-ix?

Copy and paste relevant sections from the manuscript (include quotes in quotation marks "like this" to indicate direct quotes from your manuscript), or elaborate on this item by providing additional information not in the ms, or briefly explain why the item is not applicable/relevant for your study

"The 8-week ‘My Activity Coach’ web-based intervention delivered a new module of tailored advice to participants every two weeks."

"Participants received up to four reminder emails and a reminder phone call when they didn’t complete the survey required for each module."

5-x) Clarify the level of human involvement

Clarify the level of human involvement (care providers or health professionals, also technical assistance) in the e-intervention or as co-intervention (detail number and expertise of professionals involved, if any, as well as “type of assistance offered, the timing and frequency of the support, how it is initiated, and the medium by which the assistance is delivered”. It may be necessary to distinguish between the level of human involvement required for the trial, and the level of human involvement required for a routine application outside of a RCT setting (discuss under item 21 – generalizability).

	1	2	3	4	5	
subitem not at all important	○	○	○	○	○	essential

Does your paper address subitem 5-x?

Copy and paste relevant sections from the manuscript (include quotes in quotation marks "like this" to indicate direct quotes from your manuscript), or elaborate on this item by providing additional information not in the ms, or briefly explain why the item is not applicable/relevant for your study

"Participants received up to four reminder emails and a reminder phone call when they didn’t complete the survey required for each module."

"The coaching group’s 10 to 15 minute bi-weekly video-coaching sessions were conducted through an online video-calling program of participants’ choice (e.g. Skype, Google Hangout, Yahoo Messenger and Face Time). During the session the Activity Coach commented on the tailored advice participants received in the module from the previous week, answered any questions participants had, and provided encouragement, support and accountability."

5-xi) Report any prompts/reminders used

Report any prompts/reminders used: Clarify if there were prompts (letters, emails, phone calls, SMS) to use the application, what triggered them, frequency etc. It may be necessary to distinguish between the level of prompts/reminders required for the trial, and the level of prompts/reminders for a routine application outside of a RCT setting (discuss under item 21 – generalizability).

	1	2	3	4	5	
subitem not at all important	○	○	○	○	○	essential

Does your paper address subitem 5-xi? *

Copy and paste relevant sections from the manuscript (include quotes in quotation marks "like this" to indicate direct quotes from your manuscript), or elaborate on this item by providing additional information not in the ms, or briefly explain why the item is not applicable/relevant for your study

"Participants received up to four reminder emails and a reminder phone call when they didn’t complete the survey required for each module."

5-xii) Describe any co-interventions (incl. training/support)

Describe any co-interventions (incl. training/support): Clearly state any interventions that are provided in addition to the targeted eHealth intervention, as ehealth intervention may not be designed as stand-alone intervention. This includes training sessions and support [1]. It may be necessary to distinguish between the level of training required for the trial, and the level of training for a routine application outside of a RCT setting (discuss under item 21 – generalizability.

	1	2	3	4	5	
subitem not at all important	○	○	○	○	○	essential

Does your paper address subitem 5-xii? *

Copy and paste relevant sections from the manuscript (include quotes in quotation marks "like this" to indicate direct quotes from your manuscript), or elaborate on this item by providing additional information not in the ms, or briefly explain why the item is not applicable/relevant for your study

There were no co-interventions including trailing or support.

6a) Completely defined pre-specified primary and secondary outcome measures, including how and when they were assessed

Does your paper address CONSORT subitem 6a? *

Copy and paste relevant sections from the manuscript (include quotes in quotation marks "like this" to indicate direct quotes from your manuscript), or elaborate on this item by providing additional information not in the ms, or briefly explain why the item is not applicable/relevant for your study

"Participants' demographics including gender, age, BMI, income, education, employment and location were assessed in the baseline survey. Participants were also asked if they used a video-calling program (Skype, Google hangout, Facetime, Other, None). Completion of the coaching sessions, the length of the coaching sessions and reasons for missed coaching sessions were recorded by the coach. Intervention retention and adherence were assessed by recording participants' completion of the research surveys and intervention modules respectively, and website engagement was measured through Google Analytics. Google Analytics recorded the number of website visits and time spent on the website for each participant. Intervention participants' satisfaction with the intervention was assessed at the end of the intervention (week 9). Satisfaction with module questions (4 items), computer-tailored advice (14 items), website usability (13 items), overall program (5 items) and coaching (14 items) were all assessed. The items were specifically developed for this study, though based on previous research. The items were on a 5-point Likert scale where participants were asked to rate their agreement (1=strongly agree to 5= strongly disagree) to statements about the intervention. All positively framed questions were reverse scored. For each category participants with a mean rating of 3.6 or higher (maximum = 5) were categorised as 'satisfied.' Coaching participants were also asked if they completed a coaching session and if not why not. All intervention participants were asked four open-ended questions about three topics; the advice, website and overall program. Coaching participants who completed a coaching session were asked an additional four open-ended questions specifically relating to the coaching sessions. The four questions for each category (advice, website, program and coaching) were; 1) what did you like about the advice/website/program/coaching, 2) what did you not like, 3) any recommendations for improvement and 4) any other thoughts. Responses for all questions were thematically analysed. The main outcome, weekly physical activity was assessed at all time-points by the Active Australia Questionnaire (AAQ) which is valid and reliable. Quality of life was measured at all three time points by the SF-12v2 which is valid and reliable. A physical health and a mental health component score was calculated from the SF-12v2 following manual instructions."

6a-i) Online questionnaires: describe if they were validated for online use and apply CHERRIES items to describe how the questionnaires were designed/deployed

If outcomes were obtained through online questionnaires, describe if they were validated for online use and apply CHERRIES items to describe how the questionnaires were designed/deployed [9].

	1	2	3	4	5	
subitem not at all important	○	○	○	○	○	essential

Does your paper address subitem 6a-i?

Copy and paste relevant sections from manuscript text

The SF-12 has been validated for online use however the AAQ has not. The AAQ has however been validated for self-administering, and has been delivered online in many studies:

"The main outcome, weekly physical activity was assessed at all time-points by the Active Australia Questionnaire (AAQ) which is valid [26] and reliable [27] including when self-administered [28]. Quality of life was measured at all three time points by the SF-12v2 which is valid [29] and reliable [30] including when self-administered online [31]."

CHERRIES:

Design:

"People were eligible to participate if they were English speaking Australian adults (over 18 years). Participants were excluded if they were pregnant, at risk of injury or ill health from increasing their physical activity (as assessed by the Physical Activity Readiness Questionnaire), or if they were already meeting the physical activity recommendations (as assessed via a single item asking if participants participated in 30 minutes of physical activity on most days). It is likely that the intervention attracted participants with a high internet literacy."

IRB:

"Ethics approval was received from the Central Queensland University Human Ethics Committee (H13/04-044), before recruitment took place from March 2014 to January 2015."

Development and pre-testing:

The SF-12 and AAQ has been delivered online in previous studies we have conducted. The design and programming was copied across to the current intervention website.

6a-ii) Describe whether and how "use" (including intensity of use/dosage) was defined/measured/monitored

Describe whether and how "use" (including intensity of use/dosage) was defined/measured/monitored (logins, logfile analysis, etc.). Use/adoption metrics are important process outcomes that should be reported in any ehealth trial.

1 2 3 4 5

subitem not at all important ○ ○ ○ ○ ○ essential

Does your paper address subitem 6a-ii?

Copy and paste relevant sections from manuscript text

"Completion of the coaching sessions, the length of the coaching sessions and reasons for missed coaching sessions were recorded by the coach. Intervention retention and adherence were assessed by recording participants' completion of the research surveys and intervention modules respectively, and website engagement was measured through Google Analytics. Google Analytics recorded the number of website visits and time spent on the website for each participant."

6a-iii) Describe whether, how, and when qualitative feedback from participants was obtained

Describe whether, how, and when qualitative feedback from participants was obtained (e.g., through emails, feedback forms, interviews, focus groups).

	1	2	3	4	5	
subitem not at all important	○	○	○	○	○	essential

Does your paper address subitem 6a-iii?

Copy and paste relevant sections from manuscript text

"Coaching participants were also asked if they completed a coaching session and if not why not. All intervention participants were asked four open-ended questions about three topics; the advice, website and overall program. Coaching participants who completed a coaching session were asked an additional four open-ended questions specifically relating to the coaching sessions. The four questions for each category (advice, website, program and coaching) were; 1) what did you like about the advice/website/program/coaching, 2) what did you not like, 3) any recommendations for improvement and 4) any other thoughts. Responses for all questions were thematically analysed."

6b) Any changes to trial outcomes after the trial commenced, with reasons

Does your paper address CONSORT subitem 6b? *

Copy and paste relevant sections from the manuscript (include quotes in quotation marks "like this" to indicate direct quotes from your manuscript), or elaborate on this item by providing additional information not in the ms, or briefly explain why the item is not applicable/relevant for your study

There were no changes to the trial outcomes.

7a) How sample size was determined

NPT: When applicable, details of whether and how the clustering by care provides or centers was addressed

7a-i) Describe whether and how expected attrition was taken into account when calculating the sample size

Describe whether and how expected attrition was taken into account when calculating the sample size.

	1	2	3	4	5	
subitem not at all important	○	○	○	○	○	essential

Does your paper address subitem 7a-i?

Copy and paste relevant sections from manuscript title (include quotes in quotation marks "like this" to indicate direct quotes from your manuscript), or elaborate on this item by providing additional information not in the ms, or briefly explain why the item is not applicable/relevant for your study

"Sample size calculations revealed that a sample size of 300, or 100 in each study arm, is required to detect between group differences in physical activity from baseline to post intervention using linear mixed models. This calculation was based on the alpha level of ≤0.05 (80% power) and a small effect size (.43) and 25% attrition which are common in similar interventions."

7b) When applicable, explanation of any interim analyses and stopping guidelines

Does your paper address CONSORT subitem 7b? *

Copy and paste relevant sections from the manuscript (include quotes in quotation marks "like this" to indicate direct quotes from your manuscript), or elaborate on this item by providing additional information not in the ms, or briefly explain why the item is not applicable/relevant for your study

There were no interim or stopping guidelines.

8a) Method used to generate the random allocation sequence

NPT: When applicable, how care providers were allocated to each trial group

Does your paper address CONSORT subitem 8a? *

Copy and paste relevant sections from the manuscript (include quotes in quotation marks "like this" to indicate direct quotes from your manuscript), or elaborate on this item by providing additional information not in the ms, or briefly explain why the item is not applicable/relevant for your study

"Eligible participants were randomly assigned based on a sequence (not concealed) of random numbers between 1 and 3 to one of three study arms: tailoring + video-coaching, tailoring-only or wait-list control."

8b) Type of randomisation; details of any restriction (such as blocking and block size)

Does your paper address CONSORT subitem 8b? *

Copy and paste relevant sections from the manuscript (include quotes in quotation marks "like this" to indicate direct quotes from your manuscript), or elaborate on this item by providing additional information not in the ms, or briefly explain why the item is not applicable/relevant for your study

"Eligible participants were randomly assigned based on a sequence (not concealed) of random numbers between 1 and 3 to one of three study arms: tailoring + video-coaching, tailoring-only or wait-list control. This was done in blocks of 15 participants."

9) Mechanism used to implement the random allocation sequence (such as sequentially numbered containers), describing any steps taken to conceal the sequence until interventions were assigned

Does your paper address CONSORT subitem 9? *

Copy and paste relevant sections from the manuscript (include quotes in quotation marks "like this" to indicate direct quotes from your manuscript), or elaborate on this item by providing additional information not in the ms, or briefly explain why the item is not applicable/relevant for your study

"Eligible participants were randomly assigned based on a sequence (not concealed) of random numbers between 1 and 3 to one of three study arms: tailoring + video-coaching, tailoring-only or wait-list control. This was done in blocks of 15 participants."

10) Who generated the random allocation sequence, who enrolled participants, and who assigned participants to interventions

Does your paper address CONSORT subitem 10? *

Copy and paste relevant sections from the manuscript (include quotes in quotation marks "like this" to indicate direct quotes from your manuscript), or elaborate on this item by providing additional information not in the ms, or briefly explain why the item is not applicable/relevant for your study

"SA generated the random allocation sequence and assigned participants to groups."

11a) If done, who was blinded after assignment to interventions (for example, participants, care providers, those assessing outcomes) and how

NPT: Whether or not administering co-interventions were blinded to group assignment

11a-i) Specify who was blinded, and who wasn’t

Specify who was blinded, and who wasn’t. Usually, in web-based trials it is not possible to blind the participants [1, 3] (this should be clearly acknowledged), but it may be possible to blind outcome assessors, those doing data analysis or those administering co-interventions (if any).

	1	2	3	4	5	
subitem not at all important	○	○	○	○	○	essential

Does your paper address subitem 11a-i? *

Copy and paste relevant sections from the manuscript (include quotes in quotation marks "like this" to indicate direct quotes from your manuscript), or elaborate on this item by providing additional information not in the ms, or briefly explain why the item is not applicable/relevant for your study

"It was not possible to blind researchers to participants’ group assignment after they had completed the baseline questionnaire. Participants were blinded to their group assignment only when completing their baseline questionnaire."

11a-ii) Discuss e.g., whether participants knew which intervention was the “intervention of interest” and which one was the “comparator”

Informed consent procedures (4a-ii) can create biases and certain expectations - discuss e.g., whether participants knew which intervention was the “intervention of interest” and which one was the “comparator”.

	1	2	3	4	5	
subitem not at all important	○	○	○	○	○	essential

Does your paper address subitem 11a-ii?

Copy and paste relevant sections from the manuscript (include quotes in quotation marks "like this" to indicate direct quotes from your manuscript), or elaborate on this item by providing additional information not in the ms, or briefly explain why the item is not applicable/relevant for your study

"The consent sheet explained the two interventions and therefore it is possible that participants worked out whether they were in the intervention of interest or comparator."

11b) If relevant, description of the similarity of interventions

(this item is usually not relevant for ehealth trials as it refers to similarity of a placebo or sham intervention to a active medication/intervention)

Does your paper address CONSORT subitem 11b? *

Copy and paste relevant sections from the manuscript (include quotes in quotation marks "like this" to indicate direct quotes from your manuscript), or elaborate on this item by providing additional information not in the ms, or briefly explain why the item is not applicable/relevant for your study

Not applicable

12a) Statistical methods used to compare groups for primary and secondary outcomes

NPT: When applicable, details of whether and how the clustering by care providers or centers was addressed

Does your paper address CONSORT subitem 12a? *

Copy and paste relevant sections from the manuscript (include quotes in quotation marks "like this" to indicate direct quotes from your manuscript), or elaborate on this item by providing additional information not in the ms, or briefly explain why the item is not applicable/relevant for your study

"Baseline demographics for participants in each trial arm (tailoring + video-coaching, tailoring-only and control) were presented. The demographics of completers vs. drop-outs, as well as coaching participants who did vs did not complete a coaching-session were compared using chi2 and t-tests. To test feasibility of the coaching sessions, completion of the coaching sessions, the length of the coaching sessions and reasons for missed coaching sessions were presented. Next, the two intervention groups, as well as coaching participants who did vs did not complete a coaching-session, were compared on retention (drop-outs vs completers) and adherence (completed 1-2 modules vs 3-4 modules) using a chi2-test, number of website visits and time spent on the website using t-tests and satisfaction scores (satisfied, vs neutral or not satisfied) using a chi2-test. To test effectiveness longitudinal data were analysed using intention-to-treat principles. Physical activity, mental health score and physical health score were each modelled using linear mixed models with time (baseline, week 9 and 6 months) as a repeated factor, fixed effects of time and group (control, tailoring-only, tailoring + video-coaching) and a time by group interaction. Significance level was set to p<.05."

12a-i) Imputation techniques to deal with attrition / missing values

Imputation techniques to deal with attrition / missing values: Not all participants will use the intervention/comparator as intended and attrition is typically high in ehealth trials. Specify how participants who did not use the application or dropped out from the trial were treated in the statistical analysis (a complete case analysis is strongly discouraged, and

simple imputation techniques such as LOCF may also be problematic [4]).

	1	2	3	4	5	
subitem not at all important	○	○	○	○	○	essential

Does your paper address subitem 12a-i? *

Copy and paste relevant sections from the manuscript (include quotes in quotation marks "like this" to indicate direct quotes from your manuscript), or elaborate on this item by providing additional information not in the ms, or briefly explain why the item is not applicable/relevant for your study

"Physical activity, mental health score and physical health score were each modelled using linear mixed models."

12b) Methods for additional analyses, such as subgroup analyses and adjusted analyses

Does your paper address CONSORT subitem 12b? *

Copy and paste relevant sections from the manuscript (include quotes in quotation marks "like this" to indicate direct quotes from your manuscript), or elaborate on this item by providing additional information not in the ms, or briefly explain why the item is not applicable/relevant for your study

"The demographics of completers vs. drop-outs, as well as coaching participants who did vs did not complete a coaching-session were compared using chi2 and t-tests."

"Coaching participants who did vs did not complete a coaching-session, were compared on retention (drop-outs vs completers) and adherence (completed 1-2 modules vs 3-4 modules) using a chi2-test, number of website visits and time spent on the website using t-tests and satisfaction scores (satisfied, vs neutral or not satisfied) using a chi2-test."

X26) REB/IRB Approval and Ethical Considerations [recommended as subheading under "Methods"] (not a CONSORT item)

X26-i) Comment on ethics committee approval

	1	2	3	4	5	
subitem not at all important	○	○	○	○	○	essential

Does your paper address subitem X26-i?

Copy and paste relevant sections from the manuscript (include quotes in quotation marks "like this" to indicate direct quotes from your manuscript), or elaborate on this item by providing additional information not in the ms, or briefly

explain why the item is not applicable/relevant for your study

"Ethics approval was received from the Central Queensland University Human Ethics Committee (H13/04-044), before recruitment took place from March 2014 to January 2015."

x26-ii) Outline informed consent procedures

Outline informed consent procedures e.g., if consent was obtained offline or online (how? Checkbox, etc.?), and what information was provided (see 4a-ii). See [6] for some items to be included in informed consent documents.

	1	2	3	4	5	
subitem not at all important	○	○	○	○	○	essential

Does your paper address subitem X26-ii?

Copy and paste relevant sections from the manuscript (include quotes in quotation marks "like this" to indicate direct quotes from your manuscript), or elaborate on this item by providing additional information not in the ms, or briefly explain why the item is not applicable/relevant for your study

"The consent form and then baseline questionnaire were administered through the intervention website for all groups."

X26-iii) Safety and security procedures

Safety and security procedures, incl. privacy considerations, and any steps taken to reduce the likelihood or detection of harm (e.g., education and training, availability of a hotline)

	1	2	3	4	5	
subitem not at all important	○	○	○	○	○	essential

Does your paper address subitem X26-iii?

Copy and paste relevant sections from the manuscript (include quotes in quotation marks "like this" to indicate direct quotes from your manuscript), or elaborate on this item by providing additional information not in the ms, or briefly explain why the item is not applicable/relevant for your study

Participants were given the contact details of the Central Queensland University Human Research Ethics Committee on the information sheet in case they had any concerns with the trial. Participants were also told on the information sheet that they could withdraw from the trial at anytime without any consequences.

RESULTS

13a) For each group, the numbers of participants who were randomly assigned, received intended treatment, and were analysed for the primary outcome

NPT: The number of care providers or centers performing the intervention in each group and the number of patients treated by each care provider in each center

Does your paper address CONSORT subitem 13a? *

Copy and paste relevant sections from the manuscript (include quotes in quotation marks "like this" to indicate direct quotes from your manuscript), or elaborate on this item by providing additional information not in the ms, or briefly explain why the item is not applicable/relevant for your study

"Of the 239 randomly assigned participants, 154 completed the baseline questionnaire and at least one of the intervention modules. Of these, 84 participants completed the post intervention survey at week 9 (55% retention). A total of 59 participants completed the 6 month follow up questionnaire (38% retention)."

13b) For each group, losses and exclusions after randomisation, together with reasons

Does your paper address CONSORT subitem 13b? (NOTE: Preferably, this is shown in a CONSORT flow diagram) *

Copy and paste relevant sections from the manuscript (include quotes in quotation marks "like this" to indicate direct quotes from your manuscript), or elaborate on this item by providing additional information not in the ms, or briefly explain why the item is not applicable/relevant for your study

A consort flow diagram is included with reasons for losses and exclusions.

13b-i) Attrition diagram

Strongly recommended: An attrition diagram (e.g., proportion of participants still logging in or using the intervention/comparator in each group plotted over time, similar to a survival curve) or other figures or tables demonstrating usage/dose/engagement.

	1	2	3	4	5	
subitem not at all important	○	○	○	○	○	essential

Does your paper address subitem 13b-i?

Copy and paste relevant sections from the manuscript or cite the figure number if applicable (include quotes in quotation marks "like this" to indicate direct quotes from your manuscript), or elaborate on this item by providing additional information not in the ms, or briefly explain why the item is not applicable/relevant for your study

A diagram is not included, however this information can be found in the flow diagram and in text:

"Of the 239 randomly assigned participants, 154 completed the baseline questionnaire and at least one of the intervention modules. Of these, 84 participants completed the post intervention survey at week 9 (55% retention). A total of 59 participants completed the 6 month follow up questionnaire (38% retention)."

"Just under half (47%) of participants completed at least 3 of the intervention modules."

14a) Dates defining the periods of recruitment and follow-up

Does your paper address CONSORT subitem 14a? *

Copy and paste relevant sections from the manuscript (include quotes in quotation marks "like this" to indicate direct quotes from your manuscript), or elaborate on this item by providing additional information not in the ms, or briefly explain why the item is not applicable/relevant for your study

"Recruitment took place from March 2014 to January 2015."
"The week 9 questionnaires were collected from June 2014 to April 2015."

14a-i) Indicate if critical “secular events” fell into the study period

Indicate if critical “secular events” fell into the study period, e.g., significant changes in Internet resources available or “changes in computer hardware or Internet delivery resources”

	1	2	3	4	5	
subitem not at all important	○	○	○	○	○	essential

Does your paper address subitem 14a-i?

Copy and paste relevant sections from the manuscript (include quotes in quotation marks "like this" to indicate direct quotes from your manuscript), or elaborate on this item by providing additional information not in the ms, or briefly explain why the item is not applicable/relevant for your study

No critical secular events occurred during the study period.

14b) Why the trial ended or was stopped (early)

Does your paper address CONSORT subitem 14b? *

Copy and paste relevant sections from the manuscript (include quotes in quotation marks "like this" to indicate direct quotes from your manuscript), or elaborate on this item by providing additional information not in the ms, or briefly explain why the item is not applicable/relevant for your study

The trial was not stopped early.

15) A table showing baseline demographic and clinical characteristics for each group

NPT: When applicable, a description of care providers (case volume, qualification, expertise, etc.) and centers (volume) in each group

Does your paper address CONSORT subitem 15? *

Copy and paste relevant sections from the manuscript (include quotes in quotation marks "like this" to indicate direct quotes from your manuscript), or elaborate on this item by providing additional information not in the ms, or briefly explain why the item is not applicable/relevant for your study

A table showing the baseline demographic characteristics for each group is provided.

15-i) Report demographics associated with digital divide issues

In ehealth trials it is particularly important to report demographics associated with digital divide issues, such as age, education, gender, social-economic status, computer/Internet/ehealth literacy of the participants, if known.

	1	2	3	4	5	
subitem not at all important	○	○	○	○	○	essential

Does your paper address subitem 15-i? *

Copy and paste relevant sections from the manuscript (include quotes in quotation marks "like this" to indicate direct quotes from your manuscript), or elaborate on this item by providing additional information not in the ms, or briefly explain why the item is not applicable/relevant for your study

"The majority of participants were female (76%) and were on average 54 years of age. Just under half (43%) were not employed and 83% had completed a higher education course."

"The majority (70%) used a video-calling program."

16) For each group, number of participants (denominator) included in each analysis and whether the analysis was by original assigned groups

16-i) Report multiple “denominators” and provide definitions

Report multiple “denominators” and provide definitions: Report N’s (and effect sizes) “across a range of study participation [and use] thresholds” [1], e.g., N exposed, N consented, N used more than x times, N used more than y weeks, N participants “used” the intervention/comparator at specific pre-defined time points of interest (in absolute and relative numbers per group). Always clearly define “use” of the intervention.

	1	2	3	4	5	
subitem not at all important	○	○	○	○	○	essential

Does your paper address subitem 16-i? *

Copy and paste relevant sections from the manuscript (include quotes in quotation marks "like this" to indicate direct quotes from your manuscript), or elaborate on this item by providing additional information not in the ms, or briefly explain why the item is not applicable/relevant for your study

Groups as assigned were compared on physical activity and quality of life changes, retention, adherence, engagement and satisfaction. However due to the low participation in the coaching sessions, additional analyses were conducted to compare the retention, adherence, engagement and satisfaction of the participants who competed a coaching session to all other participants.

16-ii) Primary analysis should be intent-to-treat

Primary analysis should be intent-to-treat, secondary analyses could include comparing only “users”, with the appropriate caveats that this is no longer a randomized sample (see 18-i).

	1	2	3	4	5	
subitem not at all important	○	○	○	○	○	essential

Does your paper address subitem 16-ii?

Copy and paste relevant sections from the manuscript (include quotes in quotation marks "like this" to indicate direct quotes from your manuscript), or elaborate on this item by providing additional information not in the ms, or briefly explain why the item is not applicable/relevant for your study

As explained in the methods, the primary analysis was intention to treat.

17a) For each primary and secondary outcome, results for each

group, and the estimated effect size and its precision (such as 95% confidence interval)

Does your paper address CONSORT subitem 17a? *

Copy and paste relevant sections from the manuscript (include quotes in quotation marks "like this" to indicate direct quotes from your manuscript), or elaborate on this item by providing additional information not in the ms, or briefly explain why the item is not applicable/relevant for your study

The estimate and confidence intervals of between group changes in physical activity are presented in table 2.

17a-i) Presentation of process outcomes such as metrics of use and intensity of use

In addition to primary/secondary (clinical) outcomes, the presentation of process outcomes such as metrics of use and intensity of use (dose, exposure) and their operational definitions is critical. This does not only refer to metrics of attrition (13-b) (often a binary variable), but also to more continuous exposure metrics such as “average session length”. These must be accompanied by a technical description how a metric like a “session” is defined (e.g., timeout after idle time) [1] (report under item 6a).

	1	2	3	4	5	
subitem not at all important	○	○	○	○	○	essential

Does your paper address subitem 17a-i?

Copy and paste relevant sections from the manuscript (include quotes in quotation marks "like this" to indicate direct quotes from your manuscript), or elaborate on this item by providing additional information not in the ms, or briefly explain why the item is not applicable/relevant for your study

"The average website visits and minutes spent on the website for the intervention groups was 7.53 (SD=7.14) and 87.07 (SD=77.33), respectively."

"Intervention adherence was similar for the tailoring + video-coaching group and the tailoring-only group (2= 2.13, P= 0.15)."

"Coaching participants had low adherence to the coaching sessions. Just under half of coaching group completers (48%) and under a quarter of all coaching participants (21%) completed one coaching-session. There were no demographic differences between those who participated in a coaching-session and those who did not. Of the coaching participants who did not do a coaching-session, eight wanted a second chance to do a session but did not book or show up the second time, seven refused to do one, four were too busy, seven had technical difficulties, two had injuries, and contact was lost with 13. Of those who participated in at least one coaching-session an average of 2.4 sessions were completed, the average coaching-session length was 10.4 minutes, and 15% of the coaching calls were interrupted with technical difficulties."

17b) For binary outcomes, presentation of both absolute and relative effect sizes is recommended

Does your paper address CONSORT subitem 17b? *

Copy and paste relevant sections from the manuscript (include quotes in quotation marks "like this" to indicate direct quotes from your manuscript), or elaborate on this item by providing additional information not in the ms, or briefly explain why the item is not applicable/relevant for your study

There are no binary outcomes in this intervention trial.

18) Results of any other analyses performed, including subgroup analyses and adjusted analyses, distinguishing pre-specified from exploratory

Does your paper address CONSORT subitem 18? *

Copy and paste relevant sections from the manuscript (include quotes in quotation marks "like this" to indicate direct quotes from your manuscript), or elaborate on this item by providing additional information not in the ms, or briefly explain why the item is not applicable/relevant for your study

"Participants who completed at least one coaching-session had a higher percentage of week 9 survey completers (73%), compared to other intervention participants (59%), but this difference was not significant (2= .75, P= 0.38)."

"significantly more participants who participated in the coaching sessions completed at least 3 of the intervention modules (82%) compared to other intervention participants (43%; 2= 6.03, P= .01)."

"Participants who completed the coaching sessions spent significantly longer (t (1,103) = 2.73, P=.02) on the intervention website (M=174.64, SD= 110.11) compared to other intervention participants (M=77.84, SD= 67.48), Participants who completed the coaching sessions also visited the website more frequently (M=10.20, SD= 3.85) compared to other intervention participants (M=7.25, SD= 7.36), but this difference was not significant (t (1,103) = 1.24, P= .22)."

"A higher percentage of those who participated in the coaching sessions (87.5%) were satisfied with the program compared to other participants (64.4%), however, this difference was not statistically significant (2= 1.66, P=.20)."

18-i) Subgroup analysis of comparing only users

A subgroup analysis of comparing only users is not uncommon in ehealth trials, but if done, it must be stressed that this is a self-selected sample and no longer an unbiased sample from a randomized trial (see 16-iii).

	1	2	3	4	5	
subitem not at all important	○	○	○	○	○	essential

Does your paper address subitem 18-i?

Copy and paste relevant sections from the manuscript (include quotes in quotation marks "like this" to indicate direct quotes from your manuscript), or elaborate on this item by providing additional information not in the ms, or briefly explain why the item is not applicable/relevant for your study

Due to the low participation in the coaching sessions, additional analyses were conducted to compare the retention, adherence, engagement and satisfaction of the participants who competed a coaching session to all other participants. This is presented clearly and separately to the pre-specified between group comparisons.

For example:
"Retention did not differ between intervention groups (2= 4.66, P= 0.11). Participants who completed at least one coaching-session had a higher percentage of week 9 survey completers (73%), compared to other intervention participants (59%), but this difference was not significant (2= .75, P= 0.38). Just under half (47%) of participants completed at least 3 of the intervention modules. Intervention adherence was similar for the tailoring + video-coaching group and the tailoring-only group (2= 2.13, P= 0.15), however significantly more participants who participated in the coaching sessions completed at least 3 of the intervention modules (82%) compared to other intervention participants (43%; 2= 6.03, P= .01)."

19) All important harms or unintended effects in each group

(for specific guidance see CONSORT for harms)

Does your paper address CONSORT subitem 19? *

Copy and paste relevant sections from the manuscript (include quotes in quotation marks "like this" to indicate direct quotes from your manuscript), or elaborate on this item by providing additional information not in the ms, or briefly explain why the item is not applicable/relevant for your study

"Mental health scores dropped in the tailoring-only participants at post-intervention."

19-i) Include privacy breaches, technical problems

Include privacy breaches, technical problems. This does not only include physical “harm” to participants, but also incidents such as perceived or real privacy breaches [1], technical problems, and other unexpected/unintended incidents. “Unintended effects” also includes unintended positive effects [2].

	1	2	3	4	5	
subitem not at all important	○	○	○	○	○	essential

Does your paper address subitem 19-i?

Copy and paste relevant sections from the manuscript (include quotes in quotation marks "like this" to indicate direct quotes from your manuscript), or elaborate on this item by providing additional information not in the ms, or briefly explain why the item is not applicable/relevant for your study

No privacy issues occurred.

19-ii) Include qualitative feedback from participants or observations from staff/researchers

Include qualitative feedback from participants or observations from staff/researchers, if available, on strengths and shortcomings of the application, especially if they point to unintended/unexpected effects or uses. This includes (if available) reasons for why people did or did not use the application as intended by the developers.

	1	2	3	4	5	
subitem not at all important	○	○	○	○	○	essential

Does your paper address subitem 19-ii?

Copy and paste relevant sections from the manuscript (include quotes in quotation marks "like this" to indicate direct quotes from your manuscript), or elaborate on this item by providing additional information not in the ms, or briefly explain why the item is not applicable/relevant for your study

"In response to open ended questions on the overall program participants mentioned that they liked the convenience (n= 4), ease of use (n= 4), the information (n= 5), emails (n= 2), found it motivating (n= 5), and liked the accountability (n= 2). Participants also mentioned that they would like more contact with a real person (n= 7), and thought there were too many questions (n= 2). In response to the questions on the advice, participants mentioned that it was easy to understand (n=12), was concise (n=4), laid out well (n= 4), was non-judgemental (n= 2) and liked the personalisation (n= 2). However some participants thought it was not personalised enough (n=11), did not like the online format (n=4), and learnt nothing new (n= 2). In response to open ended questions on the website, participants mentioned that the website was easy to use (n= 14) whilst others thought it was hard to use (n= 5). Lastly, some participants thought the website could use more visuals and interesting links (n =6)."

DISCUSSION

22) Interpretation consistent with results, balancing benefits and harms, and considering other relevant evidence

NPT: In addition, take into account the choice of the comparator, lack of or partial blinding, and unequal expertise of care providers or centers in each group

22-i) Restate study questions and summarize the answers suggested by the data, starting with primary outcomes and process outcomes (use)

Restate study questions and summarize the answers suggested by the data, starting with primary outcomes and process outcomes (use).

	1	2	3	4	5	
subitem not at all important	◯	◯	◯	◯	◯	essential

Does your paper address subitem 22-i? *

Copy and paste relevant sections from the manuscript (include quotes in quotation marks "like this" to indicate direct quotes from your manuscript), or elaborate on this item by providing additional information not in the ms, or briefly explain why the item is not applicable/relevant for your study

"The first aim of this study was to determine the feasibility of online video-coaching sessions. The low participation and high satisfaction with the coaching sessions suggests that the majority of people participating in web-based physical activity interventions are reluctant to talk to a coach using a video-calling program, but those who do find it worthwhile."

"The second aim of this study was to assess the effectiveness of the coaching sessions, by comparing physical activity and quality of life changes of the tailoring + video-coaching group to the tailoring only and control groups. After the 8-week intervention period there was a significant treatment effect of the tailoring + video-coaching physical activity intervention on physical activity compared to no intervention."

"The tailoring + video-coaching group improved their physical activity 27 minutes per week more in comparison to the tailoring-only group, however no significant between group differences were found."

22-ii) Highlight unanswered new questions, suggest future research

Highlight unanswered new questions, suggest future research.

	1	2	3	4	5	
subitem not at all important	◯	◯	◯	◯	◯	essential

Does your paper address subitem 22-ii?

Copy and paste relevant sections from the manuscript (include quotes in quotation marks "like this" to indicate direct quotes from your manuscript), or elaborate on this item by providing additional information not in the ms, or briefly explain why the item is not applicable/relevant for your study

"The detailed computer-tailored advice may have discouraged participants to adhere to their coaching sessions as they were satisfied with the computer-tailored advice. Core intervention content may need to be delivered in the coaching sessions to promote higher adherence. Therefore, more research is needed to determine whether online coaching is more effective than stand-alone computer-tailored advice."

20) Trial limitations, addressing sources of potential bias, imprecision, and, if relevant, multiplicity of analyses

20-i) Typical limitations in ehealth trials

Typical limitations in ehealth trials: Participants in ehealth trials are rarely blinded. Ehealth trials often look at a multiplicity of outcomes, increasing risk for a Type I error. Discuss biases due to non-use of the intervention/usability issues, biases through informed consent procedures, unexpected events.

	1	2	3	4	5	
subitem not at all important	○	○	○	○	○	essential

Does your paper address subitem 20-i? *

Copy and paste relevant sections from the manuscript (include quotes in quotation marks "like this" to indicate direct quotes from your manuscript), or elaborate on this item by providing additional information not in the ms, or briefly explain why the item is not applicable/relevant for your study

"Limitations of the research include self-reported physical activity, which may be subject to social desirability bias. Participants and researchers were not blinded to group assignment which may have biased results. The sample was predominantly female, white and educated. Therefore the results may not be generalisable to males, other cultures and low socioeconomic groups. Further, 40% of the sample was physically active at baseline despite the target group being inactive adults. This is not uncommon in physical activity studies, though results may not generalise to physically inactive Australians who are most at need of increasing their activity. The additional phone calls the coaching participants received encouraging them to complete the coaching calls may have affected their physical activity levels. However, this is expected to be minimal due to the phone discussion being focused on scheduling the coaching call rather than their physical activity. Objective measurement of physical activity (e.g. accelerometry) is needed to confirm the findings of this study. Although we were able to detect a difference in physical activity changes between the tailoring + video-coaching and tailoring only groups, the analysis was underpowered to detect other between group differences as the required sample size of 100 per group was not met. The low retention is also a significant limitation. Low retention is common in web-based programs, potentially due to the minimal face-to-face contact through either recruitment or the intervention. Similar retention rates have been observed in other web-based health behaviour interventions. Retention is likely to be even lower outside of an RCT setting which included reminder calls from researchers."

21) Generalisability (external validity, applicability) of the trial findings

NPT: External validity of the trial findings according to the intervention, comparators, patients, and care providers or centers involved in the trial

21-i) Generalizability to other populations

Generalizability to other populations: In particular, discuss generalizability to a general Internet population, outside of a RCT setting, and general patient population, including applicability of the study results for other organizations

	1	2	3	4	5	
subitem not at all important	○	○	○	○	○	essential

Does your paper address subitem 21-i?

Copy and paste relevant sections from the manuscript (include quotes in quotation marks "like this" to indicate direct

quotes from your manuscript), or elaborate on this item by providing additional information not in the ms, or briefly explain why the item is not applicable/relevant for your study

"The sample was predominantly female, white and educated. Therefore the results may not be generalisable to males, other cultures and low socioeconomic groups."

21-ii) Discuss if there were elements in the RCT that would be different in a routine application setting

Discuss if there were elements in the RCT that would be different in a routine application setting (e.g., prompts/reminders, more human involvement, training sessions or other co-interventions) and what impact the omission of these elements could have on use, adoption, or outcomes if the intervention is applied outside of a RCT setting.

	1	2	3	4	5	
subitem not at all important	○	○	○	○	○	essential

Does your paper address subitem 21-ii?

Copy and paste relevant sections from the manuscript (include quotes in quotation marks "like this" to indicate direct quotes from your manuscript), or elaborate on this item by providing additional information not in the ms, or briefly explain why the item is not applicable/relevant for your study

"Retention is likely to be even lower outside of an RCT setting which included reminder calls from researchers."

OTHER INFORMATION

23) Registration number and name of trial registry

Does your paper address CONSORT subitem 23? *

Copy and paste relevant sections from the manuscript (include quotes in quotation marks "like this" to indicate direct quotes from your manuscript), or elaborate on this item by providing additional information not in the ms, or briefly explain why the item is not applicable/relevant for your study

Trial registration: Australian New Zealand Clinical Trials Registry. ACTRN12614000339651

24) Where the full trial protocol can be accessed, if available

Does your paper address CONSORT subitem 24? *

Cite a Multimedia Appendix, other reference, or copy and paste relevant sections from the manuscript (include quotes in quotation marks "like this" to indicate direct quotes from your manuscript), or elaborate on this item by providing additional information not in the ms, or briefly explain why the item is not applicable/relevant for your study

"A detailed account of the methods can be found in the protocol paper [20]. The recruitment methods, participant eligibility, protocol, intervention description, measures and data analysis are summarised below."

25) Sources of funding and other support (such as supply of drugs), role of funders

Does your paper address CONSORT subitem 25? *

Copy and paste relevant sections from the manuscript (include quotes in quotation marks "like this" to indicate direct quotes from your manuscript), or elaborate on this item by providing additional information not in the ms, or briefly explain why the item is not applicable/relevant for your study

"Project and personal support for SA provided by the National Health and Medical Research Council (ID 1055804).
RCP is supported by a National Health and Medical Research Council Senior Research Fellowship.
CV (ID 100427) is supported by a Future Leader Fellowship from the National Heart
Foundation of Australia."

X27) Conflicts of Interest (not a CONSORT item)

X27-i) State the relation of the study team towards the system being evaluated

In addition to the usual declaration of interests (financial or otherwise), also state the relation of the study team towards the system being evaluated, i.e., state if the authors/evaluators are distinct from or identical with the developers/sponsors of the intervention.

	1	2	3	4	5	
subitem not at all important	○	○	○	○	○	essential

Does your paper address subitem X27-i?

Copy and paste relevant sections from the manuscript (include quotes in quotation marks "like this" to indicate direct quotes from your manuscript), or elaborate on this item by providing additional information not in the ms, or briefly explain why the item is not applicable/relevant for your study

"CV owns the computer-tailoring software which was used in the web-based intervention. The authors declare that there are no other conflicts of Interest."

About the CONSORT EHEALTH checklist

As a result of using this checklist, did you make changes in your manuscript? *

- yes, major changes
- yes, minor changes
- no

What were the most important changes you made as a result of using this checklist?

How much time did you spend on going through the checklist INCLUDING making changes in your manuscript *

As a result of using this checklist, do you think your manuscript has improved? *

- yes
- no
- Other:

Would you like to become involved in the CONSORT EHEALTH group?

This would involve for example becoming involved in participating in a workshop and writing an "Explanation and Elaboration" document

- yes
- no
- Other:

Any other comments or questions on CONSORT EHEALTH

STOP - Save this form as PDF before you click submit

To generate a record that you filled in this form, we recommend to generate a PDF of this page (on a Mac, simply select "print" and then select "print as PDF") before you submit it.

When you submit your (revised) paper to JMIR, please upload the PDF as supplementary file.

Don't worry if some text in the textboxes is cut off, as we still have the complete information in our database. Thank you!

Final step: Click submit !

Click submit so we have your answers in our database!

Submit

Never submit passwords through Google Forms.

Powered by
Google Forms

This content is neither created nor endorsed by Google.
Report Abuse - Terms of Service - Additional Terms
