# Supplementary figures and images for: Web-Based Video-Coaching to Assist an Automated Computer-Tailored Physical Activity Intervention for Inactive Adults: A Randomized Controlled Trial
Source: J Med Internet Res. 2016 Aug 12;18(8):e223. doi: 10.2196/jmir.5664 (PMC5002066; doi:10.2196/jmir.5664)

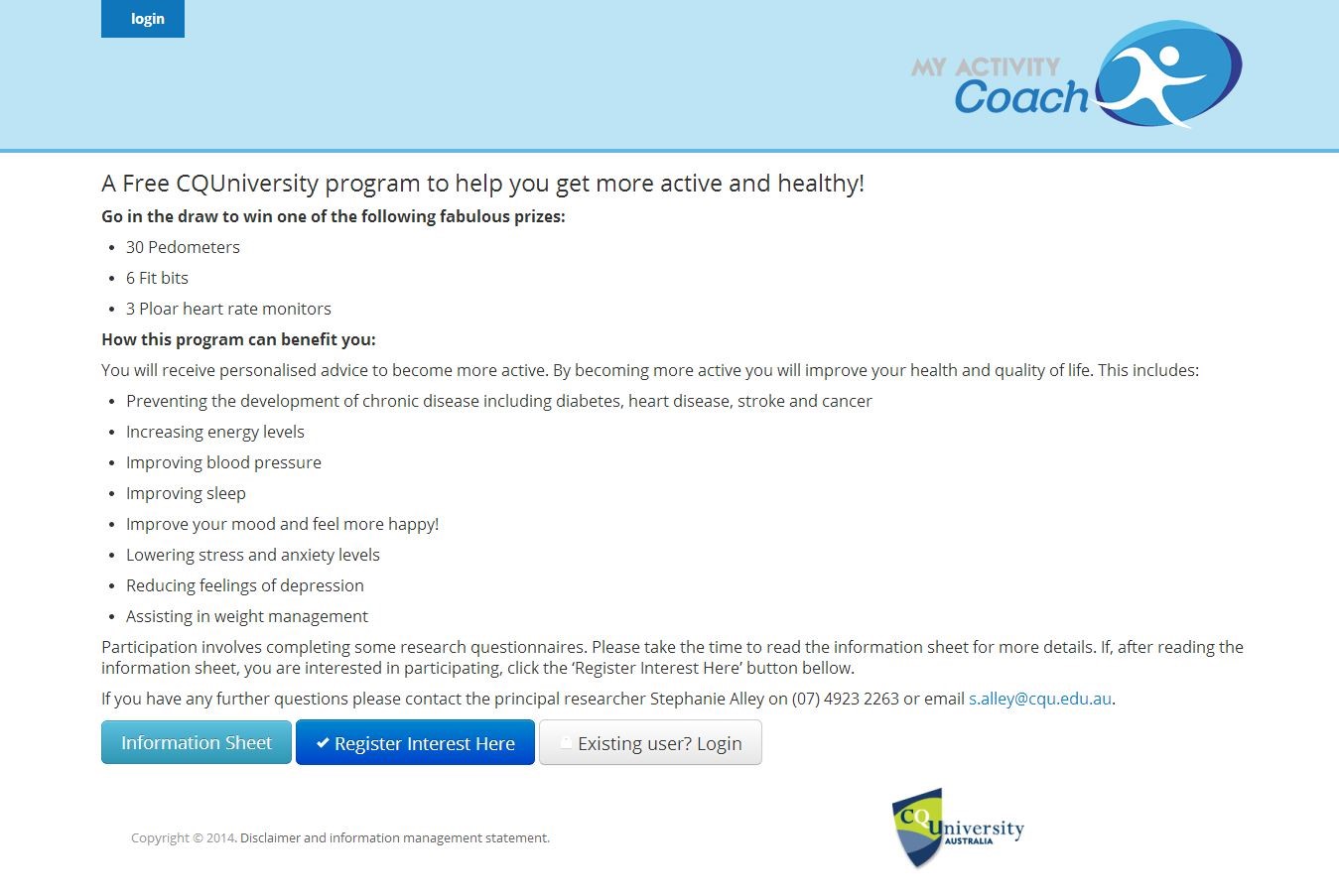

Supplement: Multimedia Appendix 2 [file jmir_v18i8e223_app2.png]

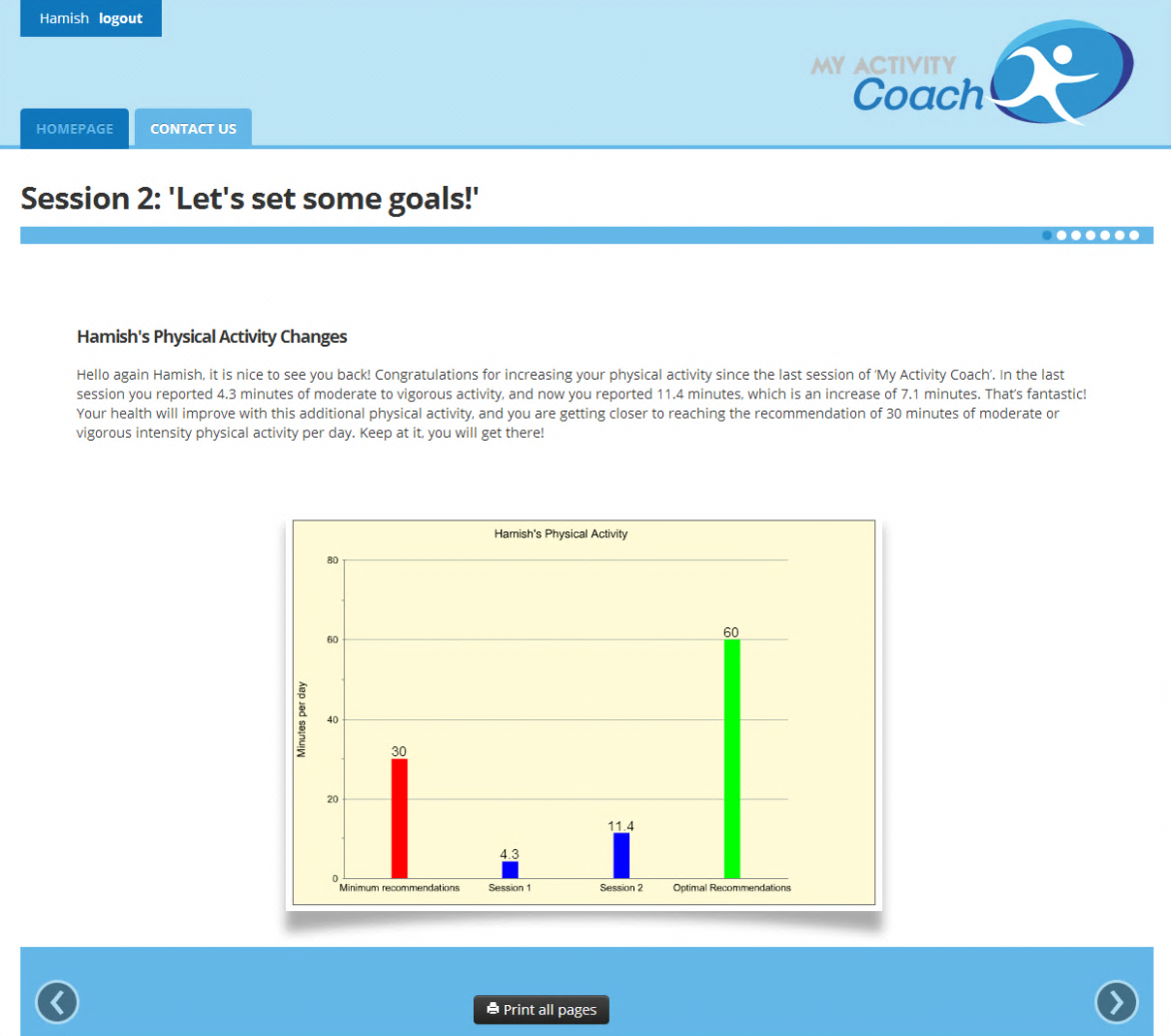

Supplement: Multimedia Appendix 4 [file jmir_v18i8e223_app4.png]

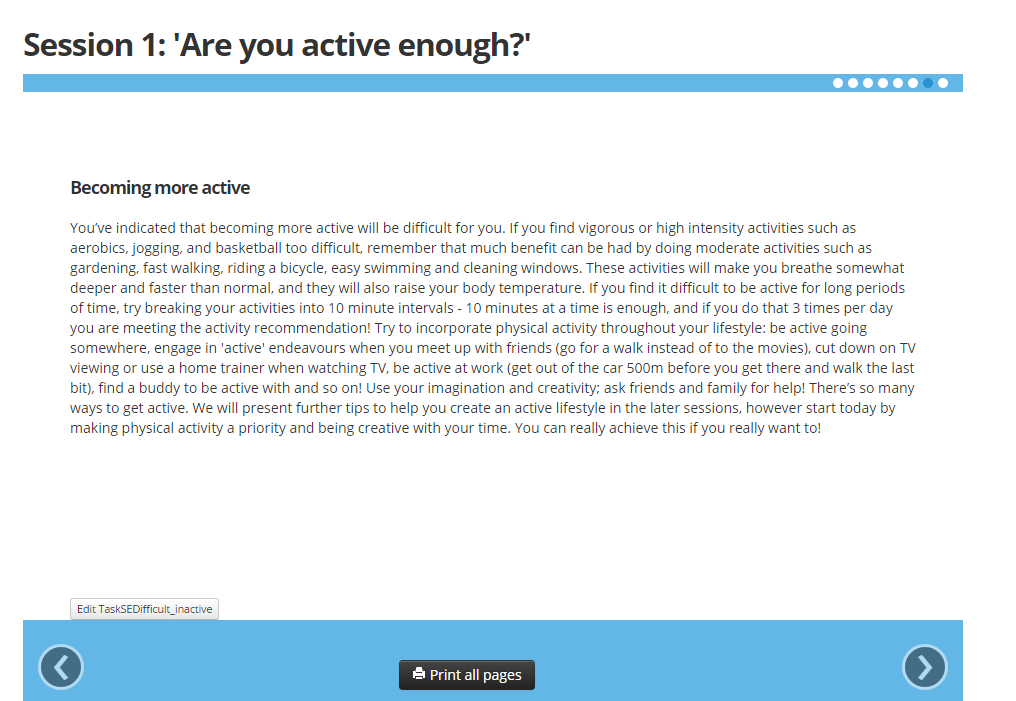

Supplement: Multimedia Appendix 5 [file jmir_v18i8e223_app5.png]

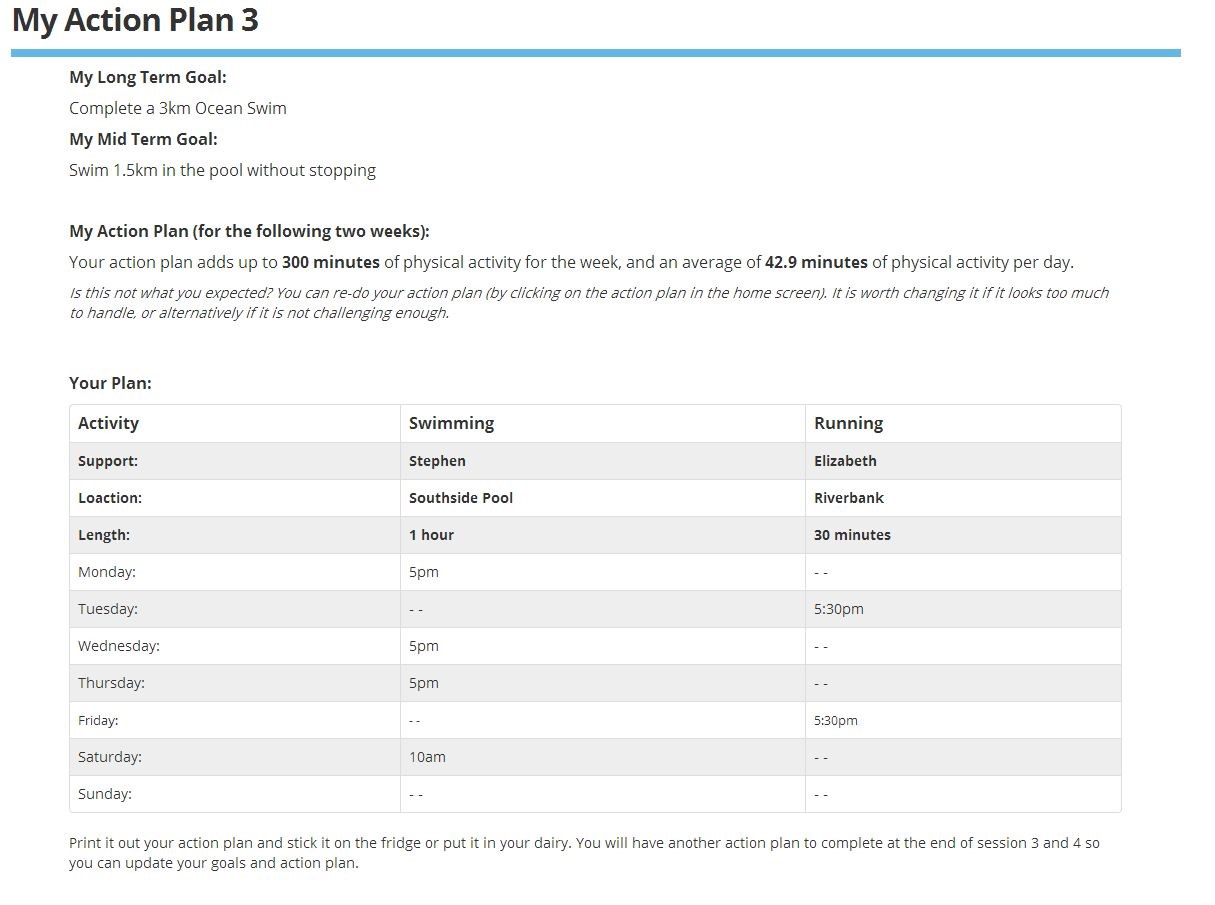

Supplement: Multimedia Appendix 6 [file jmir_v18i8e223_app6.png]
