# Supplementary material for: Web-Based Video-Coaching to Assist an Automated Computer-Tailored Physical Activity Intervention for Inactive Adults: A Randomized Controlled Trial
Source: J Med Internet Res. 2016 Aug 12;18(8):e223. doi: 10.2196/jmir.5664 (PMC5002066; doi:10.2196/jmir.5664)
Supplement: Multimedia Appendix 3 [file jmir_v18i8e223_app3.pdf]

Participant Information Form

Thank you for your interest in participating in this research. This research is being conducted in Central Queensland through the Centre for Physical Activity Studies, Central Queensland University.

The program

The program, ‘My Activity Coach’ is a state-of-the-art online physical activity program made up of four 30-minute sessions (spread over 8 weeks) that provides personalised information to help you become more active and live a healthier and happier life. Your participation in the study is entirely free.

Is this program for you?

To participate in the program you must meet **all** of the below criteria:

Speak English

Have basic computer skills, and have access to a computer with broadband Internet fast enough to watch videos directly from the internet

18 years or above

Not currently meeting the national physical activity guidelines (30 minutes of physical activity on five or more days of the week)

Have no physical impairments that will prevent you from becoming more active.

Not pregnant

Participant Commitments

Over time ALL participants will receive the physical activity program for free. In return participants are required to complete 4 online surveys (immediately prior to program commencement, and 9 weeks, 6 months and 1 year after program commencement). Each questionnaire will take approximately 20 minutes to complete.

Participants will be randomly assigned to one of 3 groups so we can establish how effective each intervention is at improving physical activity:

Video-coaching + personalised-advice: In addition to the program explained above people in this group will be asked to participate in four 10-minute coaching sessions using an internet video calling program (e.g. Skype). These coaching sessions will allow discussing the advice participants received in the program.

Personalised-advice only: People in this group will only receive the ‘My activity coach’ program as explained above.

Wait-list: People in this group will be given access to the program explained above at the end of the study after completing all surveys.

Prizes

Participants will go in the draw to win **prizes** after completing each follow-up questionnaire (9 weeks, 6 months, 1 year). The Prizes to be drawn after EACH questionnaire are: 10 pedometers, 2 Fit Bit Accelerometers, 1 Polar Heart Rate monitor.

Your rights as a participant

There are **no anticipated risks or dangers** associated with participating in this study; however the program is not appropriate for you if you have a physical impairment preventing you from becoming more active.

You are able to withdraw or decline to participate in the program at any stage with no consequences.

Any information that is obtained from participants during the program will be stored for 5 years. The **information will remain confidential** and will be stored securely. No personal information that could identify you will be disclosed in any reports on the project, or to any other party.

At the end of the study a plain English **statement summarising study findings** will be available to all participants.

Please note that there is no obligation to be involved in this program and your participation is entirely voluntary.

If you have any questions, please ask the Physical Activity Coach, Stephanie Alley.

Email: s.alley@cqu.edu.au Phone: (07) 4932 2263

Alternatively, please contact the Central Queensland University's Office of Research should there be any concerns about the nature and/or conduct of this research project.

Email: ethics@cqu.edu.au Phone: (07) 4923 2603
