# Supplementary material for: Web-Based Video-Coaching to Assist an Automated Computer-Tailored Physical Activity Intervention for Inactive Adults: A Randomized Controlled Trial
Source: J Med Internet Res. 2016 Aug 12;18(8):e223. doi: 10.2196/jmir.5664 (PMC5002066; doi:10.2196/jmir.5664)
Supplement: Multimedia Appendix 7 [file jmir_v18i8e223_app7.pdf]

Satisfaction questionnaire

Questions

The next questions are about the questions you needed to complete in order to receive the personalised physical activity advice in the 4 online sessions.

Q1. The questions were easy to understand

Strongly disagree	1	2	3	4	5	*Strongly agree*

Q2. It was easy to answer the questions

Strongly disagree	1	2	3	4	5	*Strongly agree*

Q3. There were too many questions to answer before I got the advice

Strongly disagree	1	2	3	4	5	*Strongly agree*

Q4. It’s easy to go from one question to another

Strongly disagree	1	2	3	4	5	*Strongly agree*

Advice

The next questions are about the content of the physical activity advice you received in the 4 online sessions.

Q1. The physical activity advice is interesting

Strongly disagree	1	2	3	4	5	*Strongly agree*

Q2. The physical activity advice is credible

Strongly disagree	1	2	3	4	5	*Strongly agree*

Q3. The physical activity advice is logical and well-organised

Strongly disagree	1	2	3	4	5	*Strongly agree*

Q4. The physical activity advice is easy to understand

Strongly disagree	1	2	3	4	5	*Strongly agree*

Q5. Through the physical activity advice I learned something new about my own physical activity

Strongly disagree	1	2	3	4	5	*Strongly agree*

Q6. The physical activity advice is personally relevant

Strongly disagree	1	2	3	4	5	*Strongly agree*

Q7. Too much physical activity advice was provided

Strongly disagree	1	2	3	4	5	*Strongly agree*

Q8. The physical activity advice is confusing

Strongly disagree	1	2	3	4	5	*Strongly agree*

Q9. I will use the physical activity advice

Strongly disagree	1	2	3	4	5	*Strongly agree*

Q10. The physical activity advice was persuading to become more active

Strongly disagree	1	2	3	4	5	*Strongly agree*

Q11. I have already started to become more physically active because of this program

Strongly disagree	1	2	3	4	5	*Strongly agree*

Q12. I would recommend this program to others

Strongly disagree	1	2	3	4	5	*Strongly agree*

Q13. I have changed my opinion about physical activity because of this program

Strongly disagree	1	2	3	4	5	*Strongly agree*

Q14. I would prefer to get my physical activity information from a different source

Strongly disagree	1	2	3	4	5	*Strongly agree*

Q15. What did you like about the format or content of the advice?

____

____

Q16. What did you not like about the format or content of the advice?

____

____

Q17. Do you have any recommendations for the format or content of the advice?

____

____

Q18. Do you have any other comments or suggestions about the format or content of the advice?

____

____

Brief Coaching Acceptability

The next questions are about the coaching session you participated in as part of 'My Activity Coach'.

Q1.a. Did you participate in the video-coaching sessions?

Yes

No

Some

Q1.b. (If No to Q1a) Can you briefly explain why you did not complete the video-coaching sessions?

Q2. The coaching session helped me to understand the computer-tailored advice I received in the previous week's module

Strongly disagree 1 2 3 4 5 *Strongly agree*

Q3. The coaching session helped me become more active

Strongly disagree 1 2 3 4 5 *Strongly agree*

Q4. The coaching session was enjoyable

Strongly disagree 1 2 3 4 5 *Strongly agree*

Q5. The coaching session was too short

Strongly disagree 1 2 3 4 5 *Strongly agree*

Q6. The discussions in the coaching session were relevant to me

Strongly disagree 1 2 3 4 5 *Strongly agree*

Q7. The coaching session helped me to feel supported in becoming more active

Strongly disagree 1 2 3 4 5 *Strongly agree*

Q8. I received additional information in the coaching session that helped/will help me become more active

Strongly disagree 1 2 3 4 5 *Strongly agree*

Q9. I have acted on the advice I received in the coaching session

Strongly disagree 1 2 3 4 5 *Strongly agree*

Q10. The video calling program was easy to set up on my computer

Strongly disagree 1 2 3 4 5 *Strongly agree*

Q11. The video calling program was easy to use

Strongly disagree 1 2 3 4 5 *Strongly agree*

Q12. The program would have been as helpful to me without the coaching session.

Strongly disagree 1 2 3 4 5 *Strongly agree*

Q13. The coaching session was a waste of time

Strongly disagree 1 2 3 4 5 *Strongly agree*

Q14. What did you like about the coaching sessions?

____

____

Q15. What did you not like about the coaching sessions?

____

____

Q16. Do you have any recommendations for the coaching sessions?

____

____

Q17. Do you have any other comments or suggestions about the coaching sessions?

____

____

Website Usability

The following questions are about the 'My Activity Coach' website

Q1. I think that I would like to continue to use this website

Strongly disagree 1 2 3 4 5 *Strongly agree*

Q2. I found the website unnecessarily complex

Strongly disagree 1 2 3 4 5 *Strongly agree*

Q3. I thought the website was easy to use.

Strongly disagree 1 2 3 4 5 *Strongly agree*

Q4. I was able to easily find my way around the website

Strongly disagree 1 2 3 4 5 *Strongly agree*

Q5. I found it easy to complete specific tasks on the website

Strongly disagree 1 2 3 4 5 *Strongly agree*

Q6. I liked the overall presentation of the website – colours, texts, shapes

Strongly disagree 1 2 3 4 5 *Strongly agree*

Q7. I liked the overall layout of the website – the links, tabs, how things sit on the page

Strongly disagree 1 2 3 4 5 *Strongly agree*

Q8. Navigation labels, page titles, headings and other terms were easy to understand and accurate

Strongly disagree 1 2 3 4 5 *Strongly agree*

Q9. Icons and other images were helpful and easy to understand

Strongly disagree 1 2 3 4 5 *Strongly agree*

Q10. It was easy to obtain my personal physical activity advice

Strongly disagree 1 2 3 4 5 *Strongly agree*

Q11. I found it difficult to find the information I was looking for on the website

Strongly disagree 1 2 3 4 5 *Strongly agree*

Q12. It was easy to log in and out of the website

Strongly disagree 1 2 3 4 5 *Strongly agree*

Q13. The website was very slow

Strongly disagree 1 2 3 4 5 *Strongly agree*

Q14. What did you like about using the website?

____

____

Q15. What did you not like about using the website?

____

____

Q16. Were there any problems that stopped or hindered your use of the website?

____

____

Q17. Do you have any recommendations for the website?

____

____

Q18. Do you have any other comments or suggestions about the website?

____

____

My Activity Coach Program

The following questions are about the 'My Activity Coach' program

Q1. The program gave me enough information to increase my physical activity

Strongly disagree 1 2 3 4 5 *Strongly agree*

Q2. The program was enough to help me become more active

Strongly disagree 1 2 3 4 5 *Strongly agree*

Q3. The program gave me enough support to increase my physical activity

Strongly disagree 1 2 3 4 5 *Strongly agree*

Q4. I would have liked additional components in the program

Strongly disagree 1 2 3 4 5 *Strongly agree*

Q5. The program met my expectations

Strongly disagree 1 2 3 4 5 *Strongly agree*

Q6. I wanted more from the program

Strongly disagree 1 2 3 4 5 *Strongly agree*

Q7. What did you like about the program?

____

____

Q8. What did you not like about the program?

____

____

Q9. Do you have any recommendations for the program?

____

____

Q10. Do you have any other comments or suggestions?

____

____
